# Pre-Disposition and Epigenetics Govern Variation in Bacterial Survival upon Stress

**DOI:** 10.1371/journal.pgen.1003148

**Published:** 2012-12-20

**Authors:** Ming Ni, Antoine L. Decrulle, Fanette Fontaine, Alice Demarez, Francois Taddei, Ariel B. Lindner

**Affiliations:** 1 Institut National de la Santé et de la Recherche Médicale, Paris, France; 2 Faculty of Medicine, Paris Descartes University, Paris, France; Uppsala University, Sweden

## Abstract

Bacteria suffer various stresses in their unpredictable environment. In response, clonal populations may exhibit cell-to-cell variation, hypothetically to maximize their survival. The origins, propagation, and consequences of this variability remain poorly understood. Variability persists through cell division events, yet detailed lineage information for individual stress-response phenotypes is scarce. This work combines time-lapse microscopy and microfluidics to uniformly manipulate the environmental changes experienced by clonal bacteria. We quantify the growth rates and RpoH-driven heat-shock responses of individual *Escherichia coli* within their lineage context, stressed by low streptomycin concentrations. We observe an increased variation in phenotypes, as different as survival from death, that can be traced to asymmetric division events occurring prior to stress induction. Epigenetic inheritance contributes to the propagation of the observed phenotypic variation, resulting in three-fold increase of the RpoH-driven expression autocorrelation time following stress induction. We propose that the increased permeability of streptomycin-stressed cells serves as a positive feedback loop underlying this epigenetic effect. Our results suggest that stochasticity, pre-disposition, and epigenetic effects are at the source of stress-induced variability. Unlike in a bet-hedging strategy, we observe that cells with a higher investment in maintenance, measured as the basal RpoH transcriptional activity prior to antibiotic treatment, are more likely to give rise to stressed, frail progeny.

## Author Summary

Individual organisms of identical genetic background, living in a homogeneous constant environment, may nonetheless exhibit observable differences dubbed phenotypic plasticity or variability. When such a population is challenged with an unforeseen stress, the disparity among individuals may increase, yielding different strategies in response. This work addresses the occurrence and propagation of phenotypic variation as it affects bacterial survival in response to mild antibiotic treatments. We recorded images of single bacterial cells as they divide prior to and during exposure to a sub-lethal level of streptomycin, a ribosome-targeted antibiotic. We found that individual differences increase upon stress to the extent that cells may either die or survive the treatment. Differentiation events were traced back prior to exposure. We suggest that a positive feedback loop, governed by increased membrane permeability, underlies the transient cell memory observed. Cells with relatively high basal stress-response levels prior to stress are not primed for better survival, but are rather more likely to succumb to antibiotic treatment. As pathogens commonly encounter sub-lethal doses of antibiotics, their survival may be better understood in light of this study.

## Introduction

Microbial phenotypic heterogeneity, defined as variability of a given trait in a genetically identical population in a homogeneous environment, has been repeatedly observed [1], [2]. It is manifest, for example, in the broad distributions of individual gene expression levels recorded in studies of both prokaryotic and eukaryotic cells [3], [4]. Stress conditions may induce further differentiation of clonal cells, in agreement with the observed higher variability of stress response genes' expression in comparison with other gene families [5]. At the extreme, initial stochastic variability is funneled into bistable states via positive feedback mechanisms that persist through generations [6], [7]. Recent evidence suggests that fate decisions can be partly made even before cells experience an environmental change [8], [9], [10]. A cell's ultimate fate depends on its historical state, indicating that phenotypic variability is shaped by pre-disposition factors [11].

It has been proposed that population heterogeneity increases fitness in unpredictable environments [12], [13]. This may work as a kind of bet-hedging [7], [14], [15], allowing a given genotype to express multiple phenotypes of differing viability. One phenotype may be better adapted to the current environment while others are prepared for future environmental changes under which they may gain higher fitness. On the other hand, heterogeneous populations may simply undergo performance-based selection, in which fitter cells always perform better despite an environmental change.

Stress-responsive genes show greater expression variability than genes from other classes [5], suggesting the hypothesis that variability arises as an anti-stress adaptation evolutionary strategy. Among the stresses that bacteria face, antibiotics are prominent and widespread [16]. Yet the consequences of low-grade antibiotic stress are rather poorly understood. Our interest here is to characterize the dynamic process of stress-induced phenotypic heterogeneity. Specifically, we address the following questions: Will sub-inhibitory antibiotic concentrations further amplify phenotypic variation to the extent of producing persistant and sensitive sub-populations? Are there any predetermining factors that modulate the response? How does this variation propagate through the bacterial lineage?

To this end, we followed the growth of micro-colonies from single *Escherichia. coli* (*E. coli*) cells under microfluidic control. We exposed cells to sub-inhibitory concentrations of the aminoglycoside antibiotic streptomycin and tracked their responses at the single-cell level. We find that mild antibiotic treatment results in rapid generation of increased phenotypic variability in terms of stress-induced gene expression, growth rate, survival and death. Stochastic events leading to differentiated outcomes may precede the application of stress, propagating in a more deterministic fashion within the lineage as the stress persists. Counter-intuitively, progenitors that exhibit relatively higher maintenance activity prior to stress are not primed for survival, but are rather more likely to develop frail progeny.

## Results

### Sub-inhibitory concentration of streptomycin induces heat-shock-responsive gene expression independent of genetic variability

Streptomycin penetrates aerobically growing bacteria and targets the ribosome, causing mistranslation of nascent proteins [17]. These in turn may misfold, resulting in the induction of RpoH-mediated heat-shock-responsive gene expression [18]. We monitored the heat-shock response using a chromosomal transcriptional fusion of the yellow fluorescent protein (YFP) to the RpoH-driven *ibpAB* promoter [19], [20]. This construct was found to be a highly sensitive reporter (Figure S1). We found streptomycin concentrations (<4 µg/ml), lower than the minimal inhibition concentration (MIC), where significant induction of the heat-shock response can be detected with minimal perturbation to bacterial population growth rate (Figure S2). The survival rate in these conditions, as determined by plating experiments, is 100% (see Materials and Methods).

### Stress responses vary broadly from survival to death and propagate within lineages

We followed the outcome of low-dose streptomycin treatment at the single-cell level within its lineage context by time-lapse fluorescence microscopy. This allowed us to determine the extent to which a cell's stress state depends on its ancestors and life history. From a single cell exposed to antibiotics, large variations in fluorescence and growth rate phenotypes were found to propagate through the lineage (Video S1). As can be seen in this typical movie, cells may either survive or die. As early as the first division, the two daughter cells differentiate into sub-lineages: one with higher fluorescence signal, visible inclusion bodies, slower growth and fewer total divisions before the ultimate death of all its descendants. Here ‘death’ is defined as prolonged arrest in cell growth and gradual loss of contrast in phase contrast images. The other sub-lineage grows faster (engulfing the dead cousins), exhibits lower fluorescence and further develops variation in fluorescence signal and growth rate. Periodic ‘switch on’ events, characterized by increased fluorescence and slowed growth, recur within this sub-lineage (Video S1). Thus, in response to stress induction, single cells give rise to progeny of diverse phenotypes. Other examples of stressed 2D colonies can be found in Figure S3.

We further studied the emergence of variability using a microfluidic setup allowing controlled environmental changes while following micro-colony growth with time-lapse microscopy [8]. In this setup, single cells were grown without stress for four generations prior to streptomycin treatment. The micro-colonies were monitored by phase contrast and fluorescence time-lapse microscopy (Video S2, Video S3 as representative examples). The time-series images were analyzed by our custom-made open-source software ‘Cellst’ [21] to segment the cells, quantify their growth rate and fluorescence intensities, and reconstruct their lineage (Materials and Methods).

**Figure 1:**
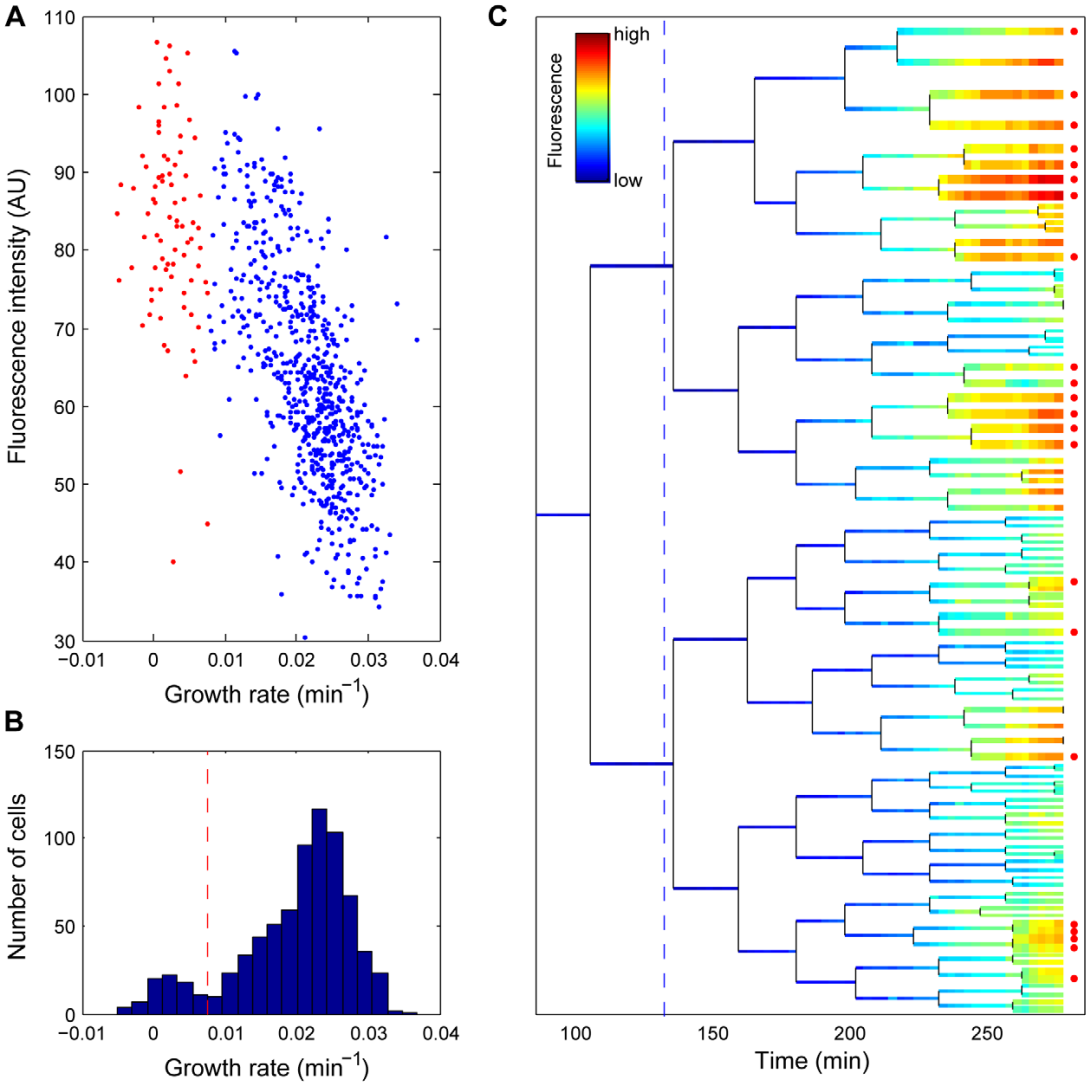
Growth inhibition and clonal cell death correlate with a high stress response. (A) Correlation between reporter intensity and growth rate in response to stress. The growth rate and fluorescence intensity of single cells are measured 130 minutes after Streptomycin treatment. Red indicates dead cells; blue, live cells. (B) Histogram of the growth rate distribution from the same data set as (A). The dashed line indicates the threshold we chose to distinguish alive from dead cells. (C) Life history of a sub-lineage from the micro-colony (see Figure 3 for the full lineage tree and the legend therein). The colour code represents the fluorescence intensity. Dead cells are indicated by a red dot at the end of the lineage tree. In addition, the cellular growth rate is represented inversely by line width (e.g., bold line the slow growers). The dashed line indicates the time of induction by streptomycin. Data correspond to Video S2.

Under induced stress conditions, the pibpAB-YFP signal was found to negatively correlate with growth rate (Figure 1A). In contrast, in absence of stress, a positive correlation prevails (Figure S4A). Therefore, the promoter fusion is a valid reporter for the protein quality, streptomycin-induced stress response. Notably, when the stress is so severe that cells stop growing, overall promoter activity diminishes. As shown in Figure 1a, the correlation saturates at low growth rates. Single cell growth rates exhibit a bimodal distribution (Figure 1B), with one sub-population identified as death-prone (Figure 1A, data points in red). The commitment to eventual cell death can be traced back as early as one generation (30 minutes, Figure S6) after induction, even though the actual death may take up to 3 generations to occur (Figure 1C, Figure S6). Staining with Propidium iodide (PI), a widely used death marker that fluoresces upon intercalation between DNA bases yet can diffuse only through depolarized cellular membranes, supports our conclusion that growth-arrested cells are indeed killed by continuous antibiotic exposure (Figure S5). While a significant (>5 hours) delay occurs between growth-arrest and PI signal, all growth-arrested cells are eventually marked.

### Phenotypic variation increases after stress

We quantified phenotypic variation as the sub-lineage coefficient of variation (SLCV) and individual coefficient of variation (IDCV) of cellular fluorescence intensity or growth rate across time (Text S1). The IDCV measures the phenotypic heterogeneity among a population of single cells regardless of their lineage relation, while the SLCV quantifies the differences among sub-populations of cells grouped according to common progenitors. For example, a single cell may divide twice to form a four-cell micro-colony. These four cells continue to divide respectively. Under normal conditions, the four subsequent sub-lineages are expected to have similar phenotypes, with relatively small differences. However, if the four sub-lineages show significantly large variation in phenotype, we would conclude that differentiation had occurred in the four-cell micro-colony, leading to significantly different sub-lineages. It follows, as depicted theoretically below, that large SLCV with respect to IDCV, indicates occurrence of differentiation.

Consider a micro-colony originated from a single cell. At time *s*, the micro-colony reaches *N_s_* cells. At a later time point *t>s*, each cell from time *s* has produced *n_i_* progeny, whose fluorescence intensity or growth rate are denoted as *x_i_^k^* (*i* = 1∼*N_s_*; *k* = 1∼*n_i_*). Therefore, the total number of cells at time *t* is *N_t_ = n_1_+n_2_…+n_Ns_*

The SLCV for a starting point *s* and end point *t* is calculated as
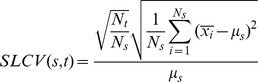
where 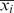 is the average phenotype among cells within the same sub-lineage and 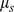 is the average across all 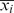 (see Text S1 for the precise definitions).

Let IDCV be the coefficient of variation among all individual cells in the micro-colony.
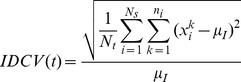
where 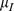 is defined as the overall average of single cell phenotypes *x_i_^k^* at given time (Text S1).

We expect that if no differentiation occurs between the sub-lineages (see Text S1 for the derivation):
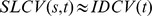
While if differentiation occurs:
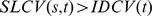
In support of this statistical model, we performed a mathematical simulation reflecting the lineage dynamics in response to the streptomycin-induced stress. A set of stochastic differential equations were constructed to describe reporter gene expression and cell division. We account for the possible positive feedback between stress level and reporter gene expression. The reporter gene expression, in turn, inversely correlates with the cellular growth rate (Figure 1A). Such feedback and correlation can lead to extended cell memory. Model parameters were set to fit the mean and variance of single cell phenotypes measured from the experimental data. As shown in Figure S7, in agreement with our expectation, the simulation results show that IDCV and SLCV are comparable in the non-stressed condition, while the extended cell memory effect leads to significantly increased SLCV in stress response.

**Figure 2:**
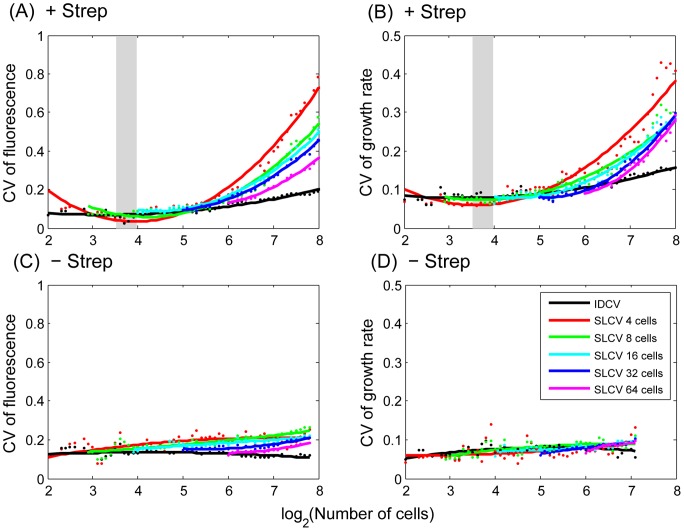
Phenotypic variation increases upon stress induction. Sub-lineage coefficient of variation (SLCV, coloured) and Individual cell coefficient of variation (IDCV, black) calculated for RpoH-driven YFP expression (A, C) and growth rate (B, D) in stressed (A, B) and non-stress (C, D) conditions (averaged from 4 micro-colonies for each condition). See main text for definition of SLCV and IDCV. Dots represent the calculated data (averaged from 4 micro-colonies) and the curves are fit with a second order polynomial function to facilitate visualization. Different colours reflect different starting points (*N_s_*) when calculating SLCV: red, 4 cells; green, 8 cells; cyan, 16 cells; blue, 32 cells; purple, 64 cells. The induction region is highlighted with grey shading.

We then calculated the actual SLCV and IDCV curves from the growth rate and fluorescence signal experimental data with different starting points *N_s_* = 4, 8, 16, 32, 64 (eg. 2∼6 generations) under induced and non-induced conditions (Figure 2). The IDCV and SLCV values are similar and stable through 8 generations of micro-colony growth without induction, indicating no differentiation (Figure 2C and 2D). In contrast, when streptomycin is added at the 8–16 cell stage, both values increase (Figure 2A and 2B). The SLCV increases faster than the IDCV, indicating differentiation. The SLCV curves with a starting point prior to induction (*N_s_* = 4, 8) also increase relative to the IDCV, indicating that differentiation potentially occurs among sibling cells even before they encounter the stress condition. This suggests that the stress has revealed a pre-existing difference in physiological states among the non-induced cells. In other words, there may exist pre-disposition factors in non-induced cells that prime the stress-induced differentiation.

We used data randomization to assess the significance of these experimental results. Randomly-chosen cells were switched within the lineage tree as follows: For a micro-colony with final population of N cells, N pairs of cells were chosen for switching to achieve sufficient mixing. Only cells born after stress induction were selected. In order to preserve the time course profile, switching was only allowed between cells of the same generation. As expected, while IDCV remains unchanged, SLCV decreases and is indistinguishable from IDCV (Figure S8). This result highlights the existence of extended memory effect in the original data.

We could exclude a genetic component to the observed variability increase under stress. Identical variability emerged by repeating the above microfluidics experiments with cells from an exponential phase culture, initially stressed (2 hours, 3 ug/ml Streptomycin), washed, and recovered for 4 hours in absence of stress (data not shown). Indeed, mutations would not be expected to reproducibly manifest these phenotypic effects given the rapid emergence of variability by 4–16 cell stage.

### The stress response is characterized by phenotypic predisposition and epigenetic inheritence

**Figure 3:**
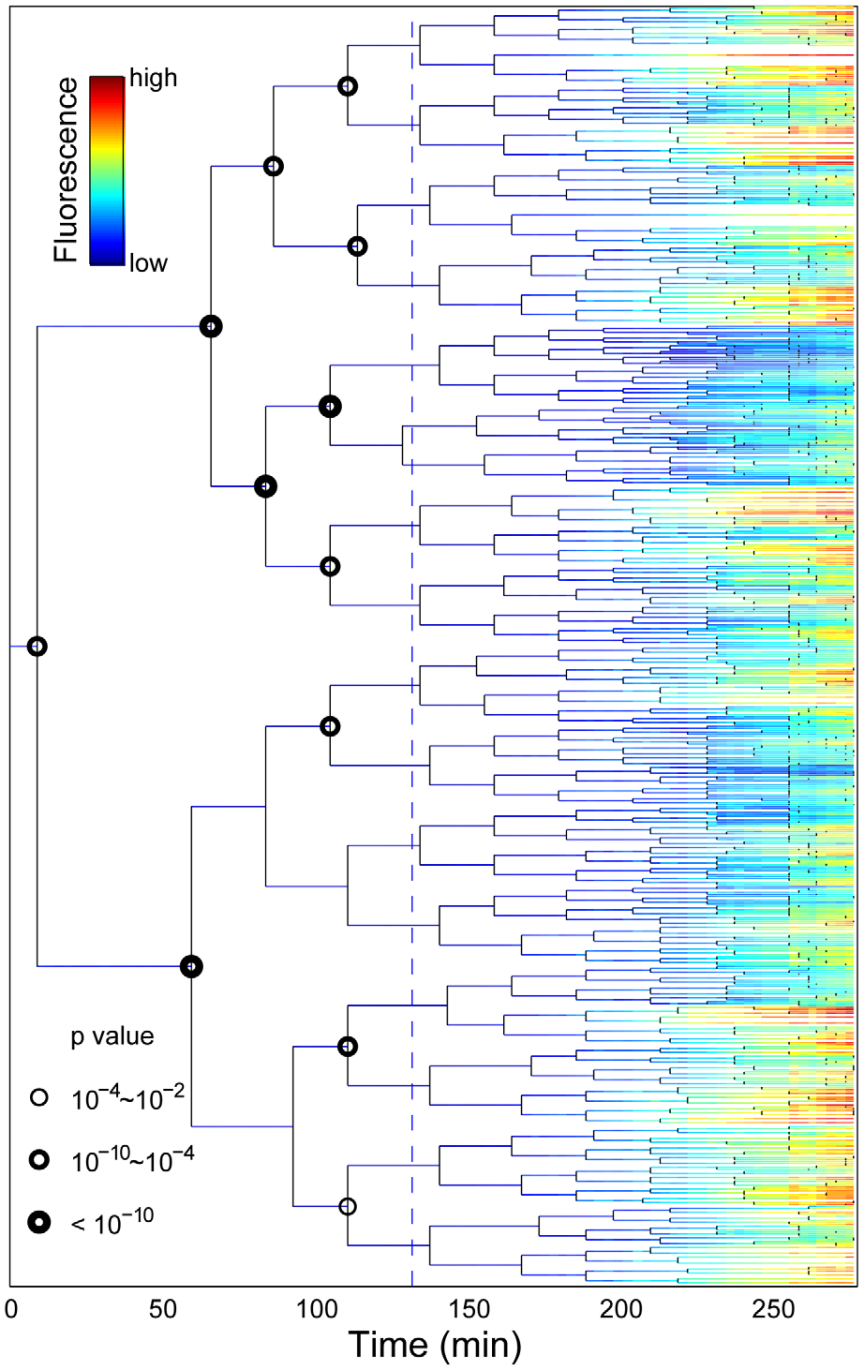
Stress-response propagation within micro-colony lineage trees reveals clustered pre-disposition events. Each horizontal line represents the life history of a single cell. The line branches when the cell divides. The fluorescence intensity of the cells is indicated by the colour code. The dashed line represents streptomycin addition. The nodes where the progeny of the sister progenitor cells have a significantly different fluorescence profiles at the end of the experiment are marked with a circle. Only nodes prior stress are considered. Different sizes of the circles indicate the p-value of T-test. Data correspond to Video S2.

In agreement with the SLCV analysis, the detailed view of the induction phenotype within the lineage context reveals significant sub-lineage divergence as well as clustering of stress induction (Figure 3; Video S2). To highlight the existence of pre-disposition factors in single cells, we compared the RpoH-driven stress response and growth rate of the descendants of each sister cell at the tree nodes prior to induction. In most cases, there was a significant difference between the mean fluorescence (T-test, p-value <0.01; circled nodes, Figure 3) and mean growth rate (Figure S10) of the two progeny groups. To assess the significance of this result, we randomly exchanged progeny measurements in the experimentally derived tree. For each pair of sister cells born prior to induction (15 nodes), we generated 500 randomized trees where progeny were randomly re-assigned. In the stark majority of the runs, no significant difference was detected between the descendants of the pre-induction sister cells. At most, fewer than two percent of the runs per node were statistically significant (p value <0.01). This is in contrast with the experimental data (Figure 3) where the majority of these events (12 of 15) are significant indicating a 15!/12!/3!)*0.02∧12 = 2E-18 probability of generating our experimental tree by chance. This suggests that differentiation between progenitor sister cells occurred prior to stress induction.

**Figure 4:**
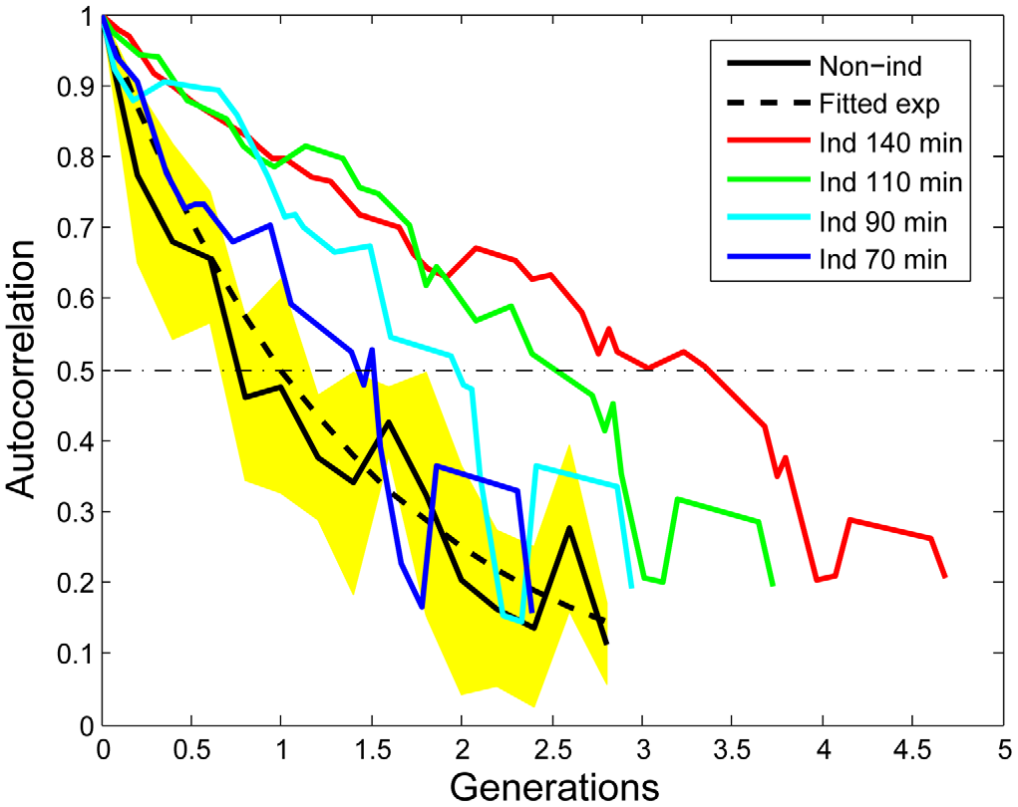
Increased auto-correlation of the stress response within micro-colonies after induction. The mathematical derivation of the auto-correlation function (AF) can be found in the Text S1. The black curve shows the AF of non-induced micro-colonies (average of 4). The yellow area indicates the standard deviation. The blue, cyan, green and red curves are the AFs calculated with starting points that are 70 minutes, 90 minutes, 110 minutes, and 140 minutes after induction respectively. The induced AF data is from the micro-colony shown in Video S2. The generation is determined by the number of cells in the micro-colony. All the curves are truncated at the 8–16 cell stage due to increased fluctuations for small sample sizes.

In search of a marker for pre-disposition, we considered differentiation events occurring within the time-scale of the stressed cellular phenotype memory half-life time (90 minutes, see below, Figure 4). That is, we compared sibling progeny at 90 minutes after induction. We found no global correlation in the comparison of fluorescence intensity, promoter activity, or growth rate between the non-induced progenitor cells and their induced progeny (Figure S9). However, for the specific identified differentiation events (T-test, p-value <0.01, Figures S11, S12, S13, S14), there is a clear bias (p-value of binomial distribution test <0.003 Figures S11, S12, S13, S14) that the more fluorescent sister gives rise to a sub-lineage with more stressed siblings.

The memory or epigenetic effect was further quantified with a gene expression level auto-correlation function. In its simplest form (i.e. stable gene product and constant production rate), this auto-correlation is expected to decrease exponentially with half-life equal to the cellular doubling time ([22]; Text S1 and Figure S19). In case of nonlinear regulation, such as a positive feedback loop, the half-life will be longer than the doubling time. To this end, we calculated the auto-correlation function of the fluorescence signal, representing the RpoH-driven gene expression level. As expected, before induction, the auto-correlation function decreases exponentially with a half-life close to the cells' doubling time (23 Minutes; Figure 4). However, a significant delay of the auto-correlation function decrease is observed after induction (Figure 4). Note that the auto-correlation function half-life increases after induction to as long as three times the cell doubling time (140 minutes, Figure 4 red line). This is indicative of epigenetic effects that last longer than a generation. It is thus likely that nonlinear effects such as positive feedback contribute to the delayed decrease in auto-correlation.

### The epigenetic inheritance of stress responses may be mediated by increased membrane permeability

It was previously proposed that streptomycin exposure could induce further streptomycin uptake by damaging the bacterial cytoplasmic (inner) membrane [23]. Such a positive feedback loop could be responsible for the epigenetic effects described above. Upon streptomycin treatment, the cytoplasmic membrane integrity is challenged by mistranslated periplasmic [23] and membrane proteins [18]. However, despite reports of increased secretion of small molecules [24], direct evidence for increased membrane permeability after streptomycin treatment is scarce. If streptomycin (molecular weight MW = 581 g/mol) treatment increases the membrane permeability, it should also increase permeability of other molecules with similar size. Therefore, controlled gene expression by transcriptional inducers such as anhydrotetracycline (ATC, analog of tetracycline, MW = 463 g/mol) should function as indicators of a parallel increase in streptomycin uptake. Similar to streptomycin, tetracycline (MW = 444 g/mol) can penetrate the outer membrane through porins [25] and diffuse across the cytoplasmic membrane. The latter step is rate limiting for both streptomycin [26] and tetracycline, with half-equilibration time of 35±15 minutes [27]. Such slow permeation rates produce detectable variation in the intracellular inducer concentration. Consider cells co-induced by streptomycin and ATC, a positive correlation between the heat-shock reporter and an ATC-inducible reporter is expected if the higher stress level induced by streptomycine leads to higher membrane permeability, with a corresponding influx of ATC molecules.

**Figure 5:**
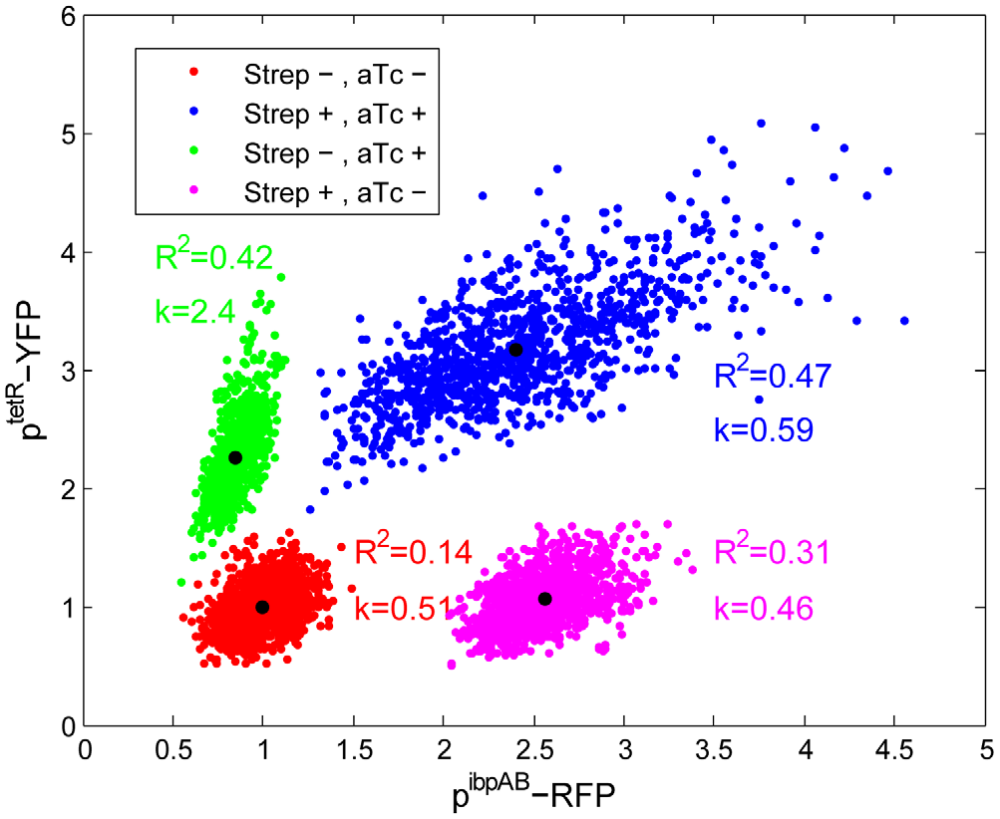
Increased cellular membrane permeability following stress induction. Exponential phase cells of a strain carrying both p*^terR^* - and p*^ibpAB^* -driven fluorescence reporters are plated on LB-agar pads with or without streptomycin (3 µg/ml) and ATC (25 ng/ml). After 2–3 hours of colony growth, the reporter intensity is quantified by fluorescence microscopy. The intensity is normalized to that of the non-induced state ([streptomycin] = [ATC] = 0). The black dot in the middle of each data cloud shows the mean value of both fluorescence channels. R^2^ and k are the coefficient of determination and slope for linear fitting. For each condition, at least 5 micro-colonies are quantified.

To test this hypothesis, a *tetR*-controlled fluorescence reporter was chromosomally integrated in the *ibpAB*-promoter-driven fluorescence reporter strain. When the strain was co-induced with both ATC and streptomycin, a positive correlation between two reporters was observed (Figure 5). Furthermore, compared to ATC induction alone, the expression level of the *tetR* reporter is stronger in the presence of streptomycin. The possibility that the positive correlation is due to elevated global protein expression level in higher stressed cells was excluded as no positive correlation was found between p*^rrna^* promoter (e.g. a constitutive promoter) and p*^ibpAB^* activity after stress (Figure S15). These results suggest that cells accumulate higher concentration of ATC under streptomycin stress, supporting the hypothesis that streptomycin stress increases the cytoplasmic membrane permeability. Such increased membrane permeability is likely to allow higher uptake of streptomycin as well, closing a positive feedback loop of stress induction which leads to the observed epigenetic effect (Figure 4). As control, we tested two other antibiotics at sub-inhibitory concentrations: Mitomycin C (a DNA cross-linker) and Nalidixic acid (topoisomerase inhibitor) that are not expected to significantly impact translational fidelity. Indeed, while these antibiotics induced the SOS response (judged by characteristic filamentation) they did not induce the ibpAB promoter and did not enhance but rather reduced the ATC induction levels (Figure S16).

## Discussion

We demonstrated that sibling *E. coli* cells diverge in their response to a sub-inhibitory concentration of streptomycin, to the extent that sub-populations may die and others survive within the same growing micro-colony in a homogeneously defined environment (Figure 1 and Figure S5). Upon induction, phenotypic differentiation events occurred, manifested as a stronger increase of the coefficient of variation among sub-lineages as compared to that of the coefficient of variation among individual cells (Figure 2), leading to significant differences between sister's progeny (Figure 3). Increased phenotypic variation upon stress is coupled with transient epigenetic inheritance that lasts for up to three generations, as opposed to a typical autocorrelation half-life of one generation time in absence of stress (Figure 4). Our results indicate the existence of nonlinear feedbacks that prolong the memory lifetime. The correlated expression of an ATC-induced *tetR* promoter and a streptomycin-induced *ibpAB* promoter (Figure 5) agrees with the hypothesis that streptomycin treatment leads to higher cellular membrane permeability, allowing more streptomycin as well as ATC molecules to enter the cell. Such feedback could be triggered by random events such as bursts of membrane damage by nascent mistranslated proteins or the asymmetric segregation of damaging factors during cell division [28]. While some cells are induced earlier and pass on the stressed state to descendants, others stay relatively healthy for a longer time, resulting in sustained diversification of cell fate. Therefore, we argue that positive feedback and stochasticity are responsible for the differentiation and increased variation.

Apart from membrane permeability, there may be other feedback pathways that can affect cell fate. For example, streptomycin may lead to production of ribosomes with lower accuracy, which in turn produce more dysfunctional ribosomes [29]. Or the amount of misfolded protein in the cell could exceed the capacity of the chaperone system, preventing the latter from maintaining protein homeostasis [30]. Recent work presents a revised view of the antibiotic mode of action, showing that apart from targeting a single entity, antibiotics broadly effect on the global metabolism of the cell [31], [32]. It is well established that sub-inhibitory concentration of antibiotics can directly or indirectly interact with different functional modules in the cell [33]. In the presence of stochastic events, such complex response processes are expected to produce diverse phenotypic outcomes. The process described here may play a role in other systems, since stochastic fluctuation and positive feedback are common. Our methodology could be applied to test other stresses, where incurred damage weakens the defense system of cells, leading to further damage accumulation.

The early occurrence of differentiation events (Figure 2 and Figure 3) indicates that the stress condition can reveal differences in cellular physiological state existing prior to induction. Some cells are intrinsically more resistant to stress than others. After induction, the difference is amplified and passed on in the respective sub-lineages, resulting in differentiation and increased variability. Similar pre-disposition phenomena have been reported in other induction-response systems. For example, the probability of lysogeny during phage infection is determined by both the number of infecting phage and the size of the host cell [9]. In the lactose switch, the bacterial growth rate and basal LacI level are highly predictive of switching outcome after induction [8]. In *Bacillus subtilis*, the decision to form an endospore is made two generations before encountering starvation conditions [10]. Whether these pre-disposition factors are a consequence of natural selection for bet hedging is mostly unclear. In the case of ampicillin ‘persisters’, a sub-population of bacteria transiently enter a dormant state in a non-stressing environment and can thus survive ampicillin treatment that attacks only growing cells [14]. Such persister cells pay a cost to express a phenotype which is less fit in the current environment but more fit for a particular environmental change. This example was interpreted as a bet-hedging strategy anticipating the arrival of future stress conditions. Yet whether it is beneficial to apply a bet-hedging strategy depends on the phenotypic switching rate, the time scale of environmental change and the fitness cost [15], or on rather stochastic events inherent to cellular physiology rather than resulting from a positive evolutionary fitness gain. In our observations, there is no sign of a pre-disposition factor working to hedge phenotypic bets. Higher stress responses prior to induction do not prime our cells for the stress to come. Instead, cells with relatively higher basal RpoH transcriptional activity are more likely to give rise to more stressed progeny (Figure S11, S12, S13, S14). This suggests that under non-stressed conditions, cells with a higher basal stress level may be paying a cost which will not help them to survive the upcoming stress. It is the weaker cells that simply suffer more, while fitter cells prevail, suggesting a simple performance-based selection.

It was recently shown that antibiotic-resistant mutants can emerge rapidly in a structured environment with a gradient of antibiotic concentrations, even from small population of 100 cells [34]. The fact that a single bacteria can generates highly variable progeny at sub-inhibitory antibiotic concentrations may facilitate this process, as it has been shown theoretically that higher variation in cellular growth rate indicates higher selection pressure [35]. Our findings may have clinical relevance as it is common that pathogens encounter sub-lethal doses of antibiotics, due either to disruptions in the prescribed medication regime or limited diffusion through structured niches such as biofilms.

## Materials and Methods

### Strains

All strains were derived from the wild-type strain *E. coli* MG1655 [36]. The YFP gene was integrated downstream of the *ibpAB* promoter with the *ibpAB* operon [19]. The strain with p*^ibpAB^*-RFP and p*^tetR^*-YFP is from [37]. The strain with p*^rrna^*-CFP and p*^ibpAB^*-RFP is from [19].

### Survival upon streptomycin stress

*E. coli* were cultured overnight at 37°C in Luria-Bertani (LB) medium (Bacto). The cell culture was diluted and plated on LB-agar plates with different concentrations of streptomycin (0–4 µg/ml). The number of colonies on the plates were counted following overnight incubation at 37°C.

### Microfluidics-microscopy setup

A detailed description of the microfluidic setup can be found in [8]. In short, cells were plated on a thin agarose pad (1.5% agarose in LB medium). The agarose pad was then inverted and laid on a cover slide with cells contacting the glass. A block of crosslinked poly(dimethylsiloxane) (PDMS RTV615, General Electric) with the feeding channel structures is exposed to air plasma (HARRICK PLASMA) and then placed over the agarose pad with the rest of surface area sticking to the cover-slide. LB medium or LB supplemented with 3 µg/ml streptomycin (Sigma) is injected into the feeding channel (2 ml per hour) and diffuses through the agarose pad to feed the cells. With this setup, it is possible to switch the medium on the spot with <1 minute homogenisation time [8].

We controlled for positional effects and no difference in cellular growth rate was found at different locations within a micro-colony (Figure S17 and S18). Additional controls on homogeneous permeability of the agarose layer have already been reported [8].

All the experiments are run at 37°C using a Zeiss automated microscope (Axio Observer Z1, HXP 120, 63× objective) with a temperature-controlled chamber (Live Imaging Services). For each media condition (with or without streptomycin), four single cells were chosen to be followed. Phase contrast photos were taken every 90 seconds while fluorescence photos were taken every 180 seconds (2% lamp energy, 3 second exposure).

### 2D micro-colony growth on agarose pad

Overnight cultures in LB 37°C were diluted 200 fold into fresh LB and agitated at 37°C for 2 hours. 1 µl of cell culture was dropped onto an agarose pad (1.5% agarose in LB medium with or without 3 µg/ml streptomycin or 25 ng/ml ATC). The agarose pad was covered with a cover-slide and the border sealed with nail polish [38], [39].

### Image analysis and lineage reconstruction

Phase contrast images were analyzed by customized software “Cellst” [21] for cell segmentation and micro-colony lineage reconstruction. The cell border was then projected onto the corresponding fluorescence image to determine the fluorescence intensity of the cells, defined as the mean grey level (background subtracted) of the pixels inside cell border. The exact location of a cell was set as the pixel coordinate of the centre of mass of the cell area. The length of a cell is measured as the long axis of the cell area.

## Supporting Information

Figure S1 *E. coli* Population growth in presence of low streptomycin concentrations. Overnight culture is diluted 200 fold into fresh medium and agitated in 37°C for 2 hours. The culture is then diluted into medium with 0–4 µg/ml of streptomycin respectively. Further dilution is performed two hour afterwards to maintain low cell-density (OD<0.3) for measurement accuracy. In each treatment, 1 ml of sample is taken for OD measurement every hour until 4 hours after induction. Cell population is calculated as the OD value multiplied by the dilution factor, then normalized by the initial value at time zero.
(PDF)

Figure S2 Flow cytometry quantification of single cell p*^ibpAB^* driven fluorescence expression after streptomycin induction. For sample preparation see the legend of Figure S1. In each treatment, 1 ml of sample is taken every hour and kept on ice. Four hours after stress induction, all the samples are measured by flow-cytometry (Becton-Dickinson FACSAria) for YFP expression.
(PDF)

Figure S3 Micro-colonies grown in the presence of streptomycin. Overnight culture is recovered in fresh medium for 2 hours then plated onto LB-agar with 3 µg/ml streptomycin. The images are taken 250 minutes after plating. The upper row is phase contrast image and the bottom is fluorescence image for the *ibpAB* promoter driven YFP expression. In these representative images we can see a great variation and clustered pattern of fluorescence intensity and co-occurrence of high fluorescence signal and inclusion body.
(PDF)

Figure S4 Correlation between cell growth rate and fluorescence intensity. (A) Non-induced condition. The growth rate and fluorescence signal are measured two hours after inoculation into microfluidics device. (B) 60 minutes after stress induction. (C) 90 minutes after stress induction. Four micro-colonies are quantified in each condition.
(PDF)

Figure S5 Streptomycin induced cell death characterized by Propidium Iodide (PI) staining. Cells were inoculated to agar pad with 4 ug/ml streptomycin and 10 ug/ml PI. The upper row shows the phase contrast snapshots of colony growth and the lower lane shows the red fluorescence signal from DNA intercalation of PI of depolarized cells. Note that grey-level scale is different between the first three images (to reflect background staining of growing cells) and the last four images (scaled such that the highly fluorescent cells will not blur the whole colony, fully-stained in the last image).
(PDF)

Figure S6 Location of dead cells in lineage tree. Dead cells and their common ancestors are high-lighted in red. The blue dashed line indicates the time of streptomycin induction. Dead cells are identified according to Figure 1B. The green dashed box indicates the sub-lineage shown in Figure 1C.
(PDF)

Figure S7 Stochastic modeling of IDCV and SLCV. The different colours of lines represent the same measures as in Figure 2, which are the mean value and the 99.8% confidence region of the mean (three fold of standard error) from 100 times of independent runs. (A) without stress induction; (B) with stress induction.
(PDF)

Figure S8 Lineage structure randomization decreases SLCV. (A) The coefficient of measured variation of cellular fluorescence (data from the same lineage as in main text Figure 1, Figure 3) (B) 250 pairs of cells are chosen for switching their position in the lineage tree. Only cells that are born after stress induction are selected. Switch only happens between cells in the same generation. While IDCV remains unchanged, SLCV decrease to indistinguishable to IDCV. The mean value and the 99.8% confidence region of the mean (three fold of standard error) are presented.
(PDF)

Figure S9Phenotypic correlations between non-induced cells and their stress induced progenies. (A) Fluorescence intensity. (B) Promoter activity, calculated as 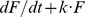, where F is fluorescence intensity and k is cellular growth rate (C) Growth rate. All the values are normalized by the mean of respective micro-colony. Four micro-colonies are quantified.(PDF)

Figure S10 Pre-disposition clustered events detected by cellular growth rate. The lineage tree is same as Figure 3. The nodes where the two progenies emanating from two respective sister progenitor cells have a statistical significant difference at the end of the experiment in terms of growth rate are marked by a circle.
(PDF)

Figure S11 Correlation between progenitor sister cells fluorescence and progenies fluorescence intensity. The lineage tree corresponds to Figure 3 but with shorter time scale until 90 minutes after induction. When the significant event highlighted in red, the higher fluorescent progenitor sister gives rise to a sub-lineage with higher stress level. Otherwise it is highlighted in blue.
(PDF)

Figure S12 Correlation between progenitor sister cells fluorescence and progenies fluorescence intensity. The lineage tree corresponds to Video S3. Experimental condition is the same as Figure S11.
(PDF)

Figure S13 Correlation between progenitor sister cells fluorescence and progenies fluorescence intensity. Experimental condition is the same as Figure S11.
(PDF)

Figure S14 Correlation between progenitor sister cells fluorescence and progenies fluorescence intensity. Experimental condition is the same as Figure S11.
(PDF)

Figure S15 Correlation between p^ipbAB^ promoter and p^rrna^ promoter activity. Overnight culture is diluted 200 fold in LB in 37°C for 2 hours. The exponential phase cell culture is then diluted into LB medium with or without streptomycin for another 2 hour. Cells are then plated on agar pad and quantified under fluorescence microscope. Red and blue dots indicate non-induced and induced cells respectively. Fluorescence levels are normalized by the mean value of non-induced cells. The black dots indicate the mean value.
(PDF)

Figure S16 Co-induction of antibiotics and aTc. Exponential phase cells of strain harbouring both p*^terR^* and p*^ibpAB^* driven fluorescence reporters are plated onto LB-agar pads containing ATC (25 ng/ml) with or without (A) Mitomycin (0.3 µg/ml) and (B) Nalidixic Acid (0.6 µg/ml). After 2–3 hours of colony growth, the fluorescence intensity is quantified under fluorescence microscope. The fluorescence intensity is normalized by the non-induced state ([mitoycin] = [nalidixic acid] = [ATC] = 0). The black dot in the middle of each data cloud shows the mean value of both fluorescence channels. For each condition, at least 5 micro-colonies are quantified.
(PDF)

Figure S17 Cellular distance from micro-colony border. The distance between a single cell and the boarder of the micro-colony (BMC) is calculated as follows. Cells that are at the BMC are identified as boarder cells. The distance between boarder cells to BMC is zero. For the non-boarder cells, the distance of the cell to BMC is defined as the minimum distance between this cell to every boarder cells (pixel distance between cell mass centre). In this way, as long as the geometry of the micro-colony is not too far from convex polygon, which is mostly the case in our experiments, such definition is a good approximate of the geographical state of cells. In this figure, the dots represent mass centre of each cell. The colour denotes the BMC value. This is extracted from the last image of Video S2.
(PDF)

Figure S18 Correlation between cellular growth rate and their geographic location. The cellular growth rate is found to be independent of the distance of the cell to the boarder of micro-colony. Four micro-colonies (Figures S11, S12, S13, S14) are quantified at the last images of the time-lapsed movies.
(PDF)

Figure S19Autocorrelation half-life determined by the linear model. The autocorrelation half-life (relative to the cellular doubling time) is calculated according to equation (4) for different values of 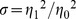 and *t*.(PDF)

Text S1 Supplementary Methods.
(PDF)

Video S1 Time-lapse movie of micro-colony growth on 2D agar pad with 3 ug/ml streptomycin. The fluorescence signal is p*^ibpAB^* driven YFP expression.
(MOV)

Video S2 Time-lapse movie of micro-colony growth in microfluidics chamber with streptomycin induction at fourth generation.
(MOV)

Video S3 Time-lapse movie of micro-colony growth in microfluidics chamber with streptomycin induction at fourth generation.
(MOV)
