# Supplementary figures and images for: Pre-Disposition and Epigenetics Govern Variation in Bacterial Survival upon Stress
Source: PLoS Genet. 2012 Dec 20;8(12):e1003148. doi: 10.1371/journal.pgen.1003148 (PMC3527273; doi:10.1371/journal.pgen.1003148)

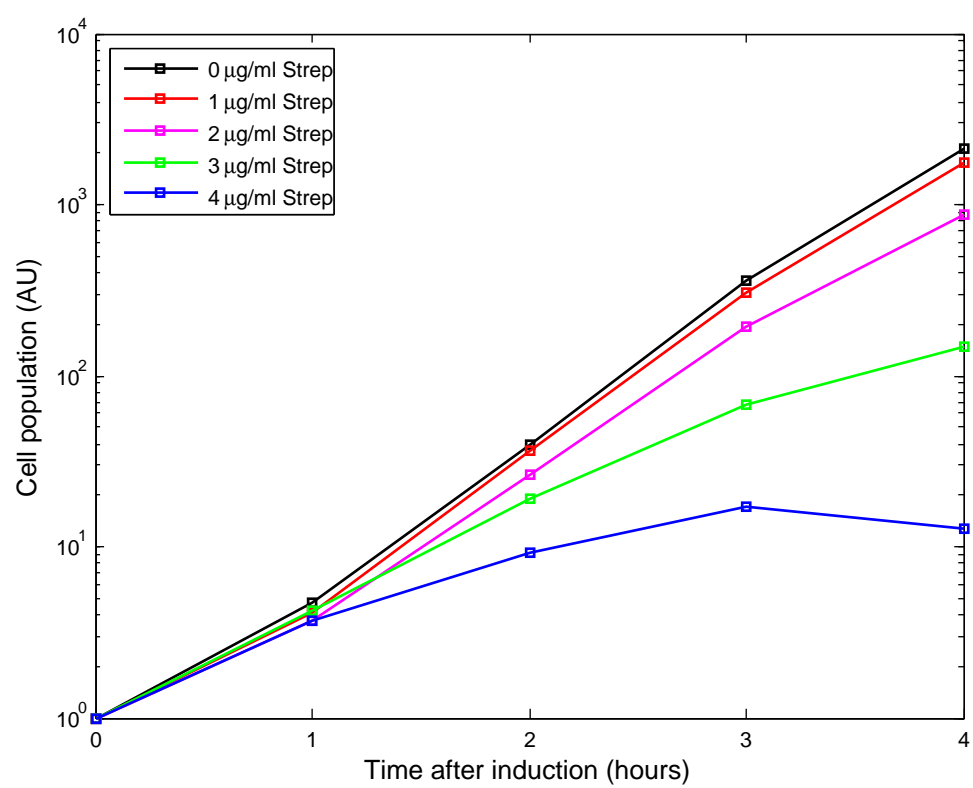

Supplement: Figure S1 — E. coli Population growth in presence of low streptomycin concentrations. Overnight culture is diluted 200 fold into fresh medium and agitated in 37°C for 2 hours. The culture is then diluted into medium with 0–4 µg/ml of streptomycin respectively. Further dilution is performed two hour afterwards to maintain low cell-density (OD<0.3) for measurement accuracy. In each treatment, 1 ml of sample is taken for OD measurement every hour until 4 hours after induction. Cell population is calculated as the OD value multiplied by the dilution factor, then normalized by the initial value at time zero. (PDF) [file pgen.1003148.s001.pdf]

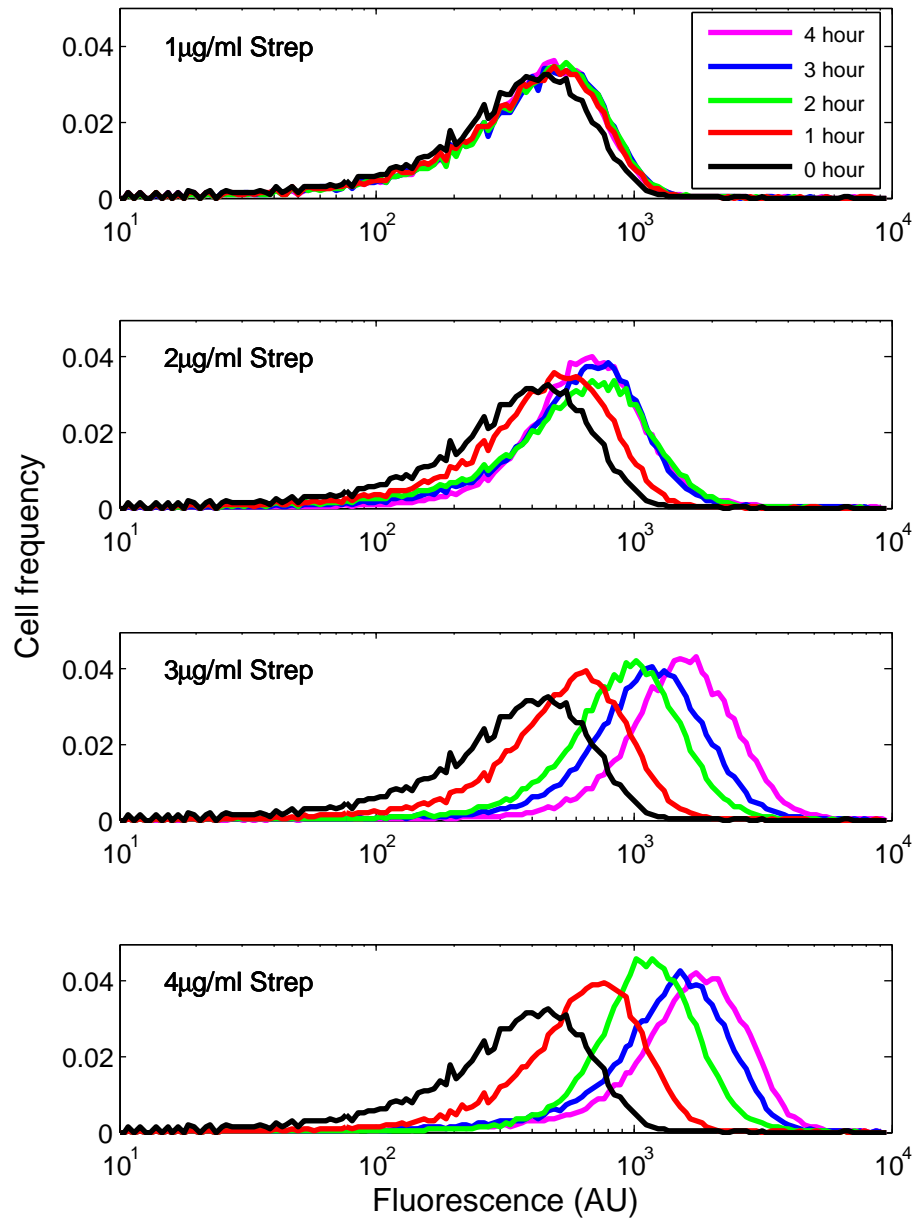

Supplement: Figure S2 — Flow cytometry quantification of single cell pibpAB driven fluorescence expression after streptomycin induction. For sample preparation see the legend of Figure S1. In each treatment, 1 ml of sample is taken every hour and kept on ice. Four hours after stress induction, all the samples are measured by flow-cytometry (Becton-Dickinson FACSAria) for YFP expression. (PDF) [file pgen.1003148.s002.pdf]

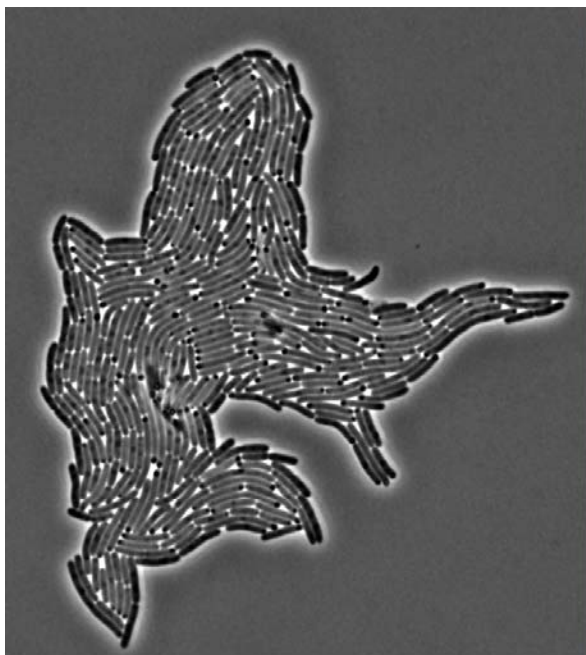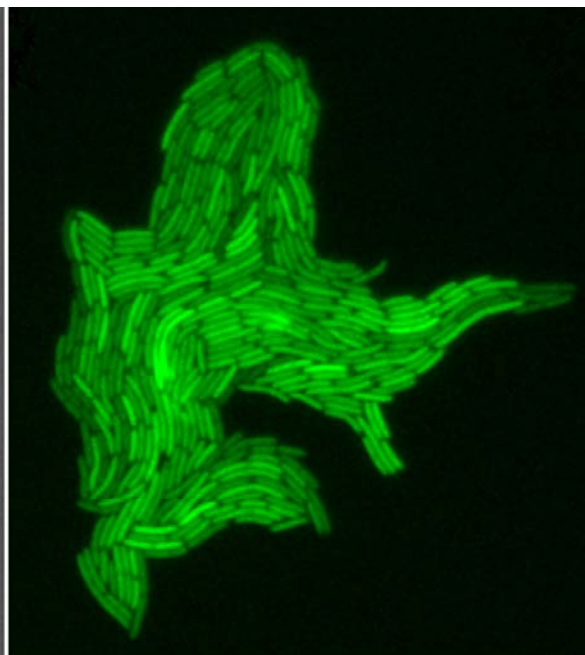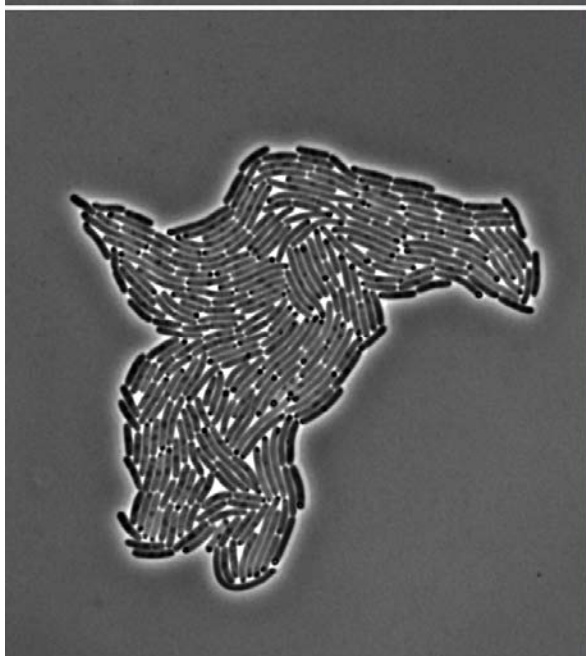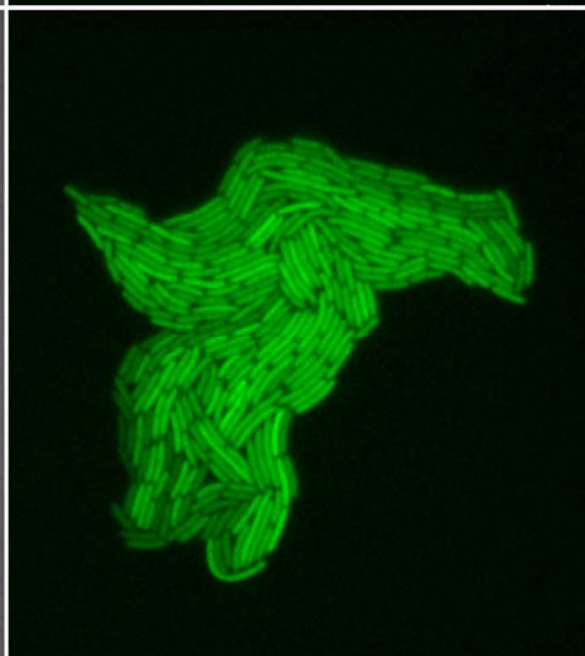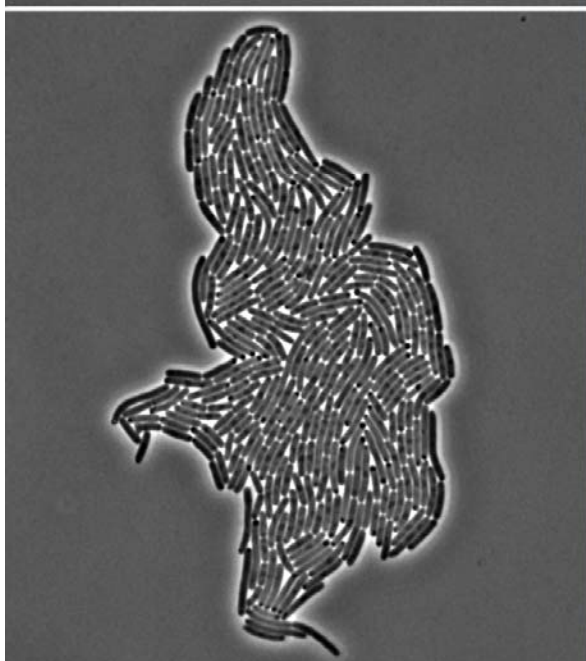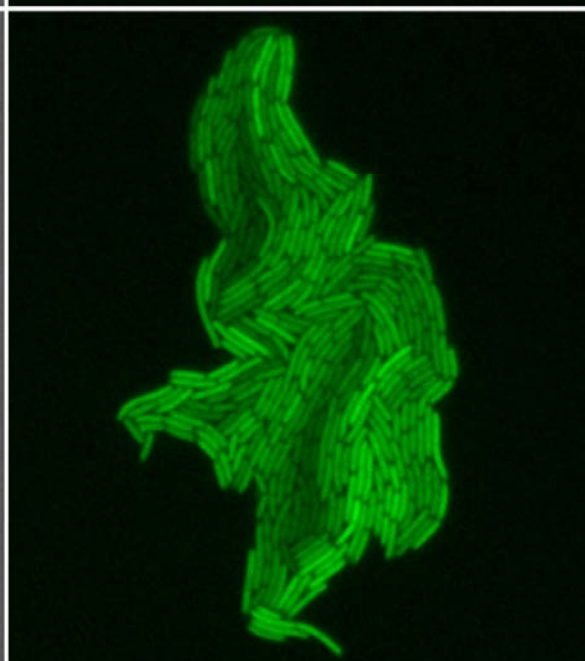

Supplement: Figure S3 — Micro-colonies grown in the presence of streptomycin. Overnight culture is recovered in fresh medium for 2 hours then plated onto LB-agar with 3 µg/ml streptomycin. The images are taken 250 minutes after plating. The upper row is phase contrast image and the bottom is fluorescence image for the ibpAB promoter driven YFP expression. In these representative images we can see a great variation and clustered pattern of fluorescence intensity and co-occurrence of high fluorescence signal and inclusion body. (PDF) [file pgen.1003148.s003.pdf]

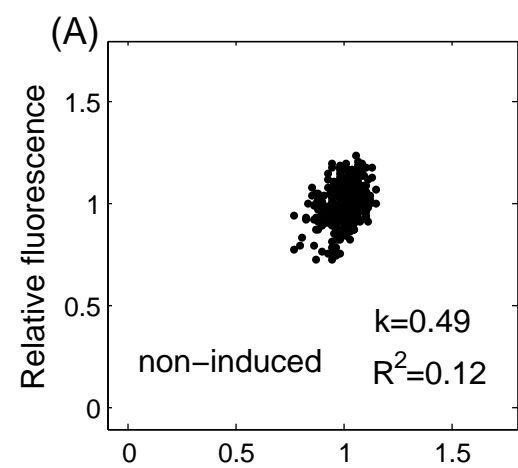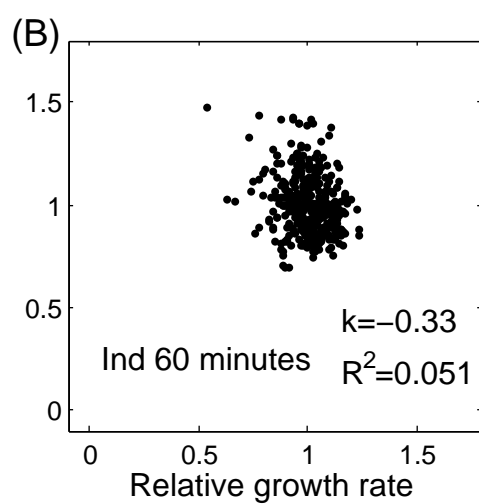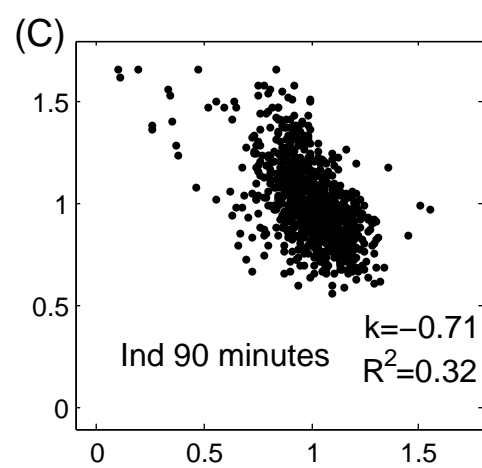

Supplement: Figure S4 — Correlation between cell growth rate and fluorescence intensity. (A) Non-induced condition. The growth rate and fluorescence signal are measured two hours after inoculation into microfluidics device. (B) 60 minutes after stress induction. (C) 90 minutes after stress induction. Four micro-colonies are quantified in each condition. (PDF) [file pgen.1003148.s004.pdf]

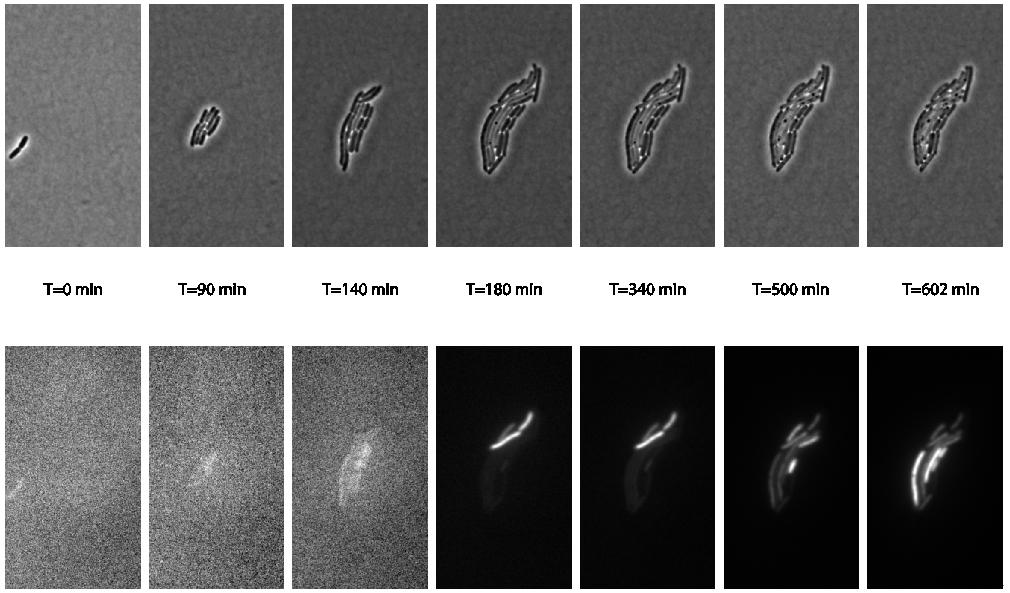

Supplement: Figure S5 — Streptomycin induced cell death characterized by Propidium Iodide (PI) staining. Cells were inoculated to agar pad with 4 ug/ml streptomycin and 10 ug/ml PI. The upper row shows the phase contrast snapshots of colony growth and the lower lane shows the red fluorescence signal from DNA intercalation of PI of depolarized cells. Note that grey-level scale is different between the first three images (to reflect background staining of growing cells) and the last four images (scaled such that the highly fluorescent cells will not blur the whole colony, fully-stained in the last image). (PDF) [file pgen.1003148.s005.pdf]

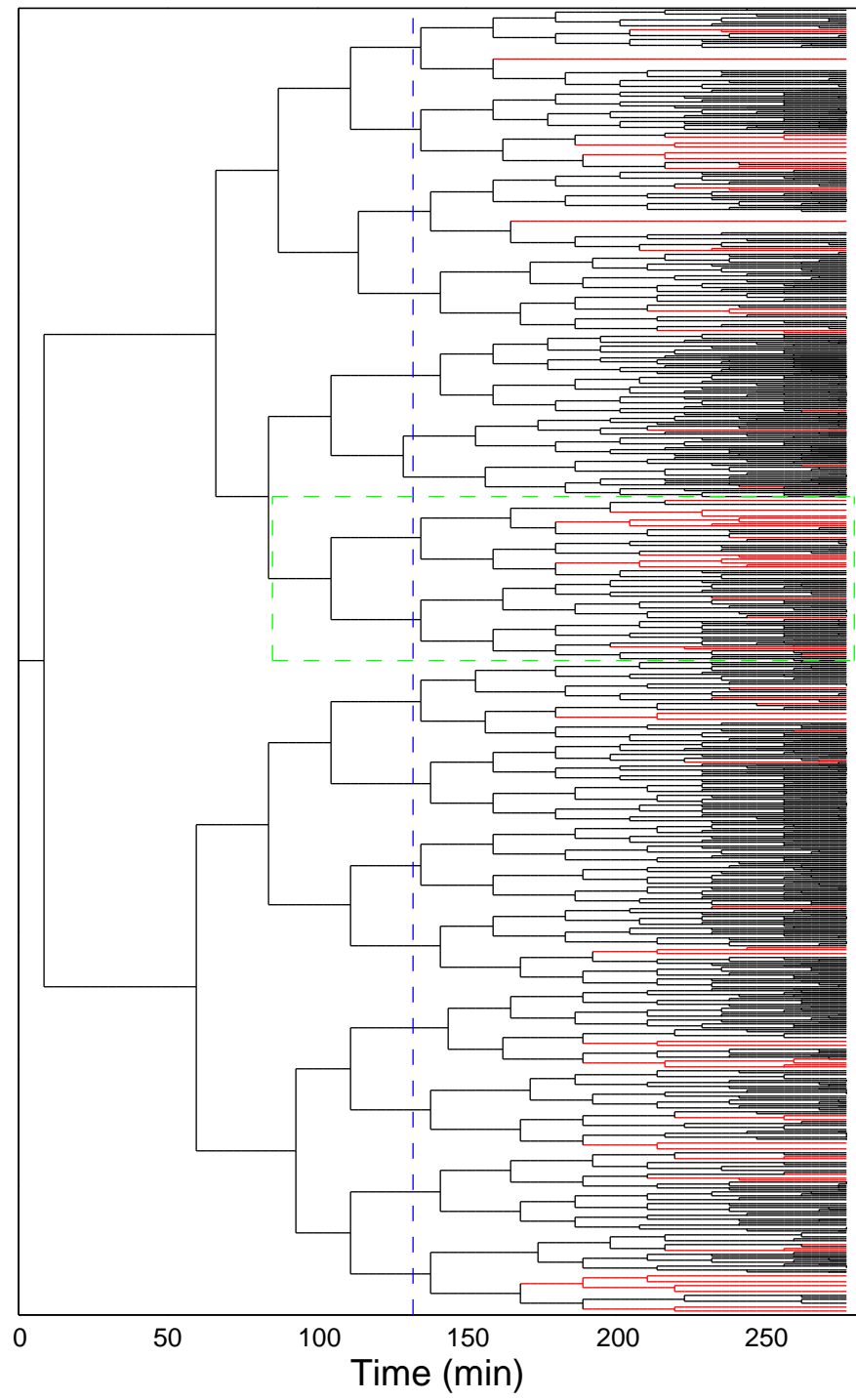

Supplement: Figure S6 — Location of dead cells in lineage tree. Dead cells and their common ancestors are high-lighted in red. The blue dashed line indicates the time of streptomycin induction. Dead cells are identified according to Figure 1B. The green dashed box indicates the sub-lineage shown in Figure 1C. (PDF) [file pgen.1003148.s006.pdf]

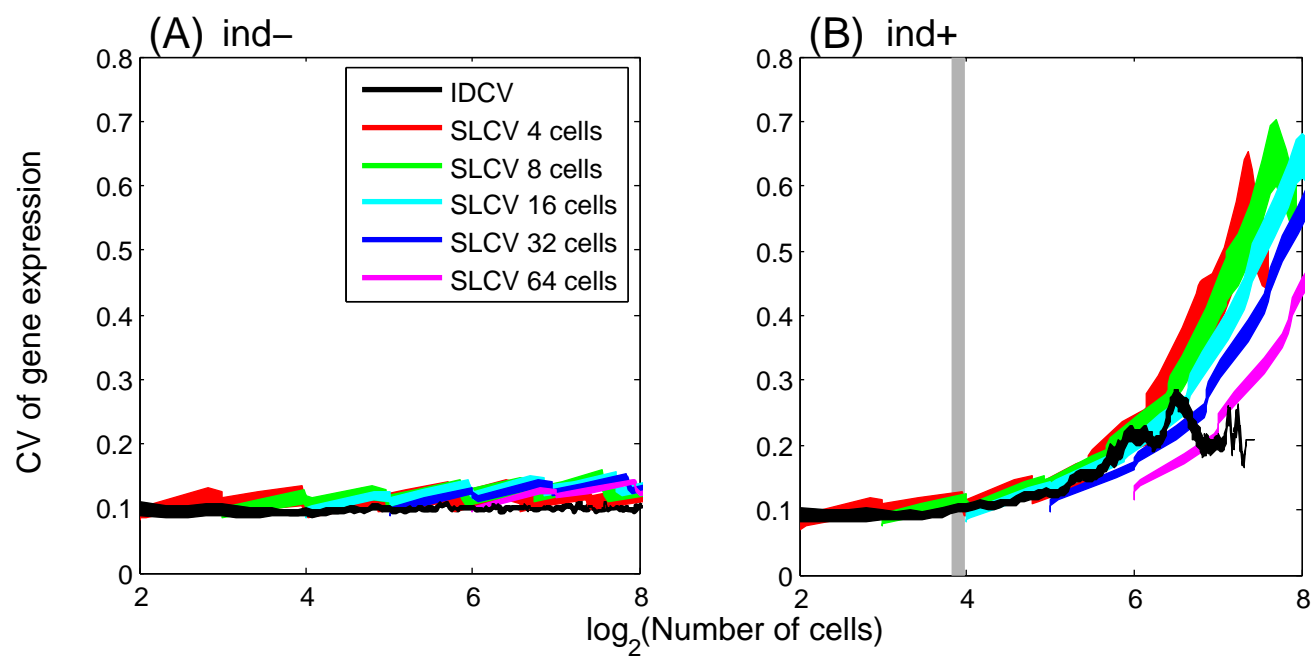

Supplement: Figure S7 — Stochastic modeling of IDCV and SLCV. The different colours of lines represent the same measures as in Figure 2, which are the mean value and the 99.8% confidence region of the mean (three fold of standard error) from 100 times of independent runs. (A) without stress induction; (B) with stress induction. (PDF) [file pgen.1003148.s007.pdf]

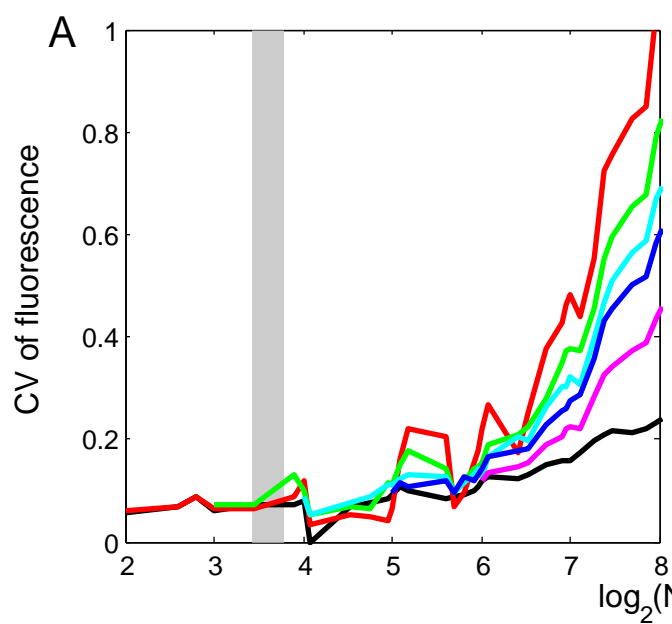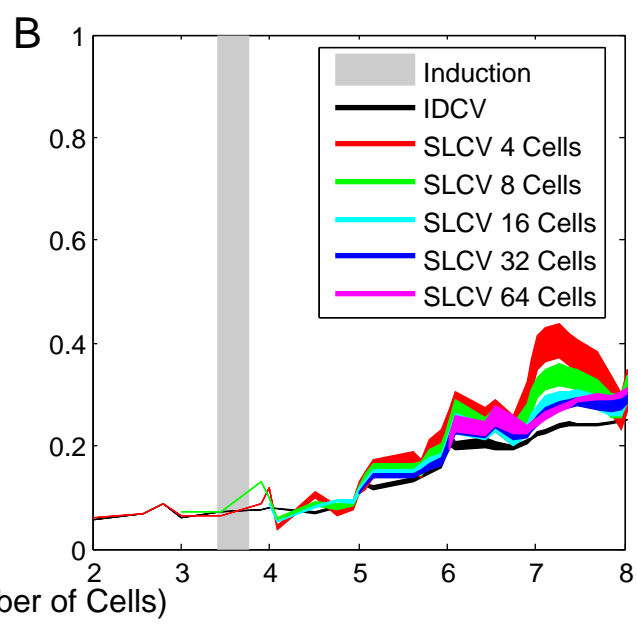

Supplement: Figure S8 — Lineage structure randomization decreases SLCV. (A) The coefficient of measured variation of cellular fluorescence (data from the same lineage as in main text Figure 1, Figure 3) (B) 250 pairs of cells are chosen for switching their position in the lineage tree. Only cells that are born after stress induction are selected. Switch only happens between cells in the same generation. While IDCV remains unchanged, SLCV decrease to indistinguishable to IDCV. The mean value and the 99.8% confidence region of the mean (three fold of standard error) are presented. (PDF) [file pgen.1003148.s008.pdf]

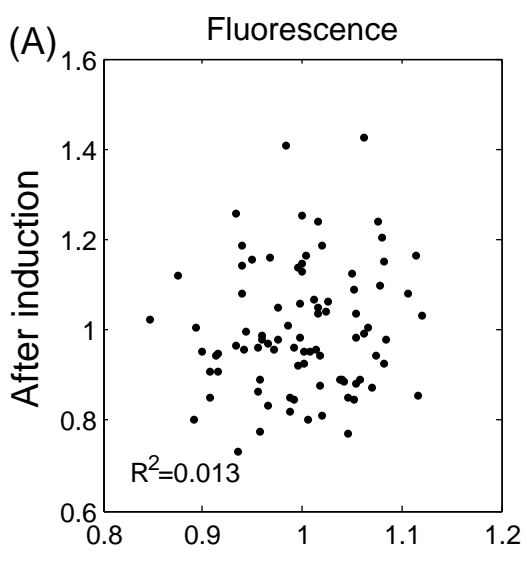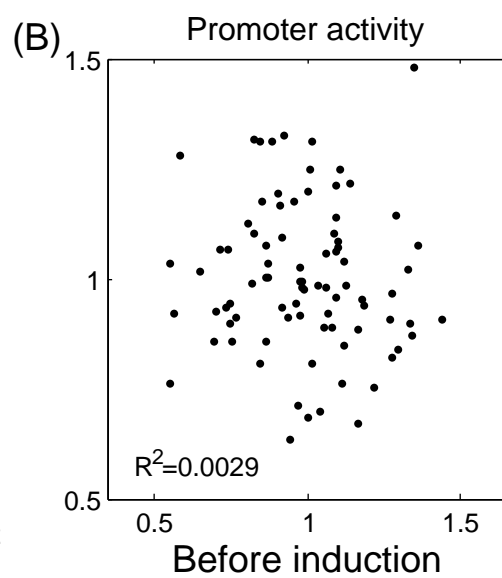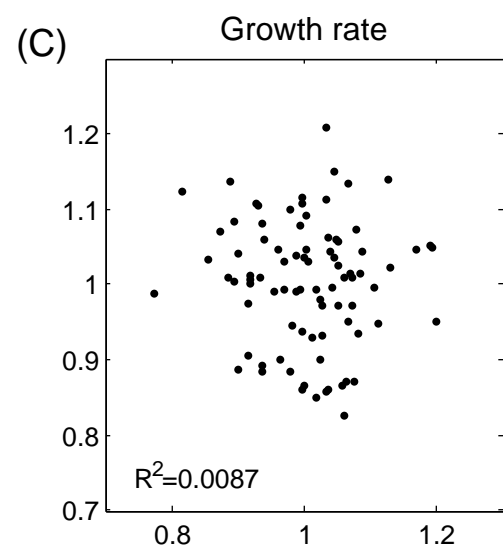

Supplement: Figure S9 — Phenotypic correlations between non-induced cells and their stress induced progenies. (A) Fluorescence intensity. (B) Promoter activity, calculated as , where F is fluorescence intensity and k is cellular growth rate (C) Growth rate. All the values are normalized by the mean of respective micro-colony. Four micro-colonies are quantified. (PDF) [file pgen.1003148.s009.pdf]

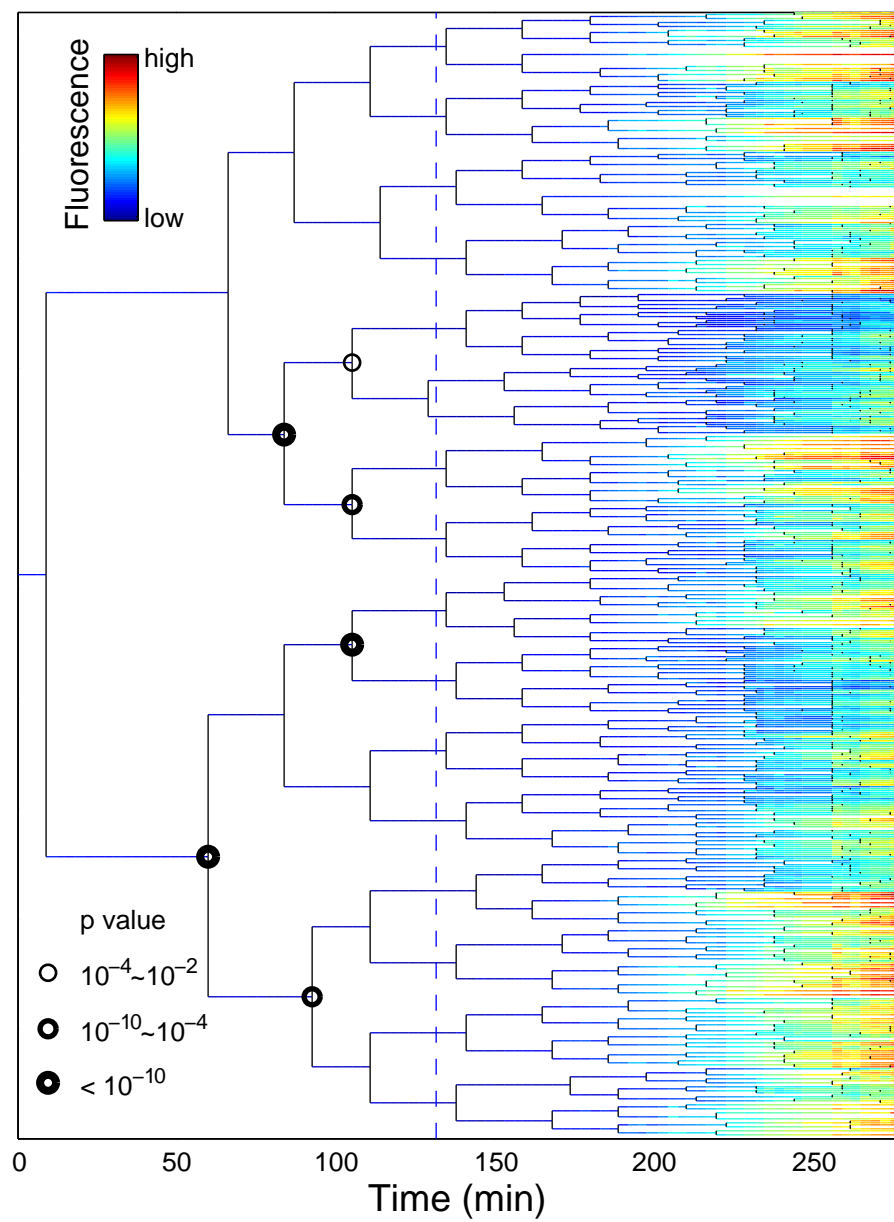

Supplement: Figure S10 — Pre-disposition clustered events detected by cellular growth rate. The lineage tree is same as Figure 3. The nodes where the two progenies emanating from two respective sister progenitor cells have a statistical significant difference at the end of the experiment in terms of growth rate are marked by a circle. (PDF) [file pgen.1003148.s010.pdf]

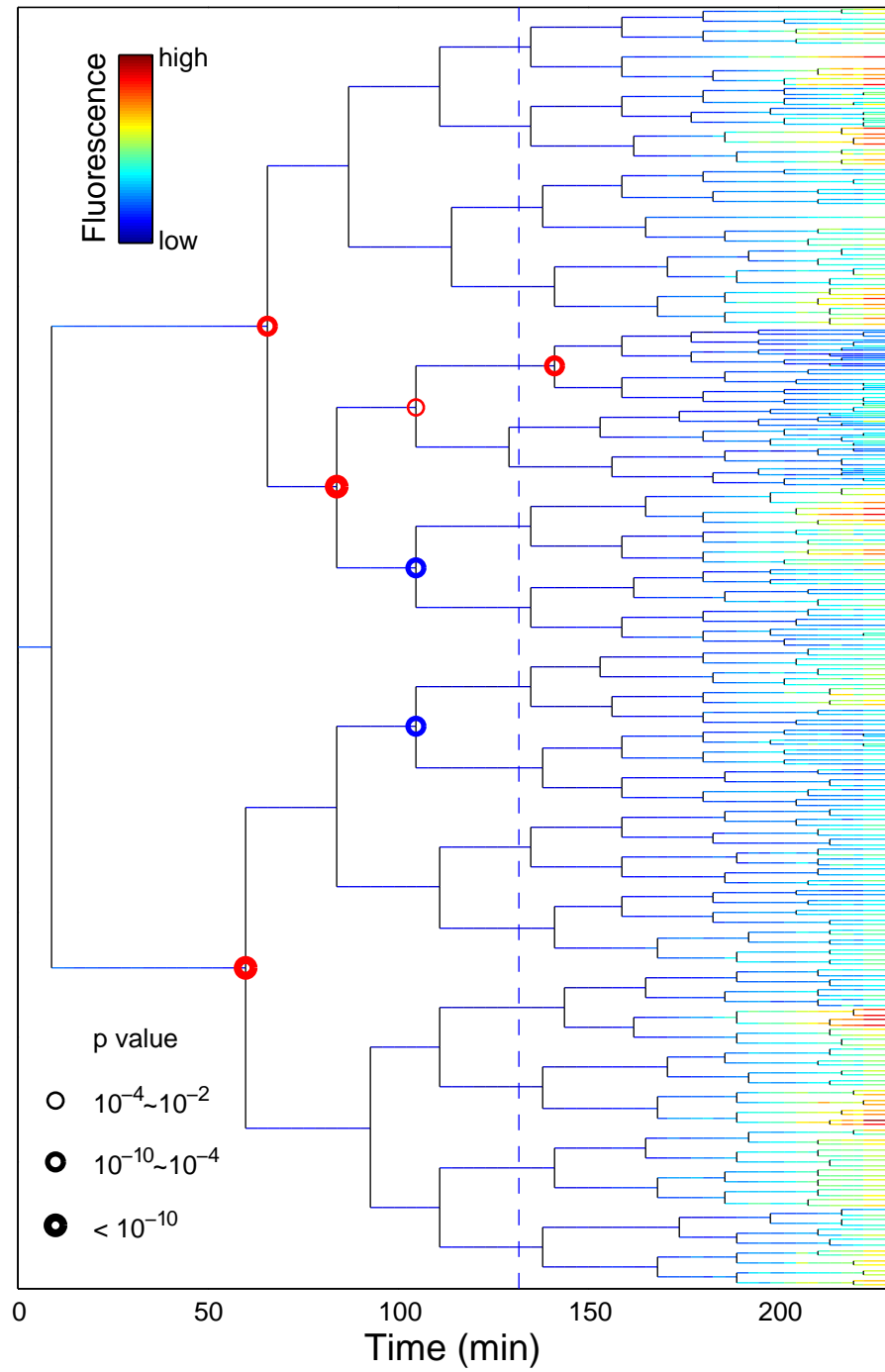

Supplement: Figure S11 — Correlation between progenitor sister cells fluorescence and progenies fluorescence intensity. The lineage tree corresponds to Figure 3 but with shorter time scale until 90 minutes after induction. When the significant event highlighted in red, the higher fluorescent progenitor sister gives rise to a sub-lineage with higher stress level. Otherwise it is highlighted in blue. (PDF) [file pgen.1003148.s011.pdf]

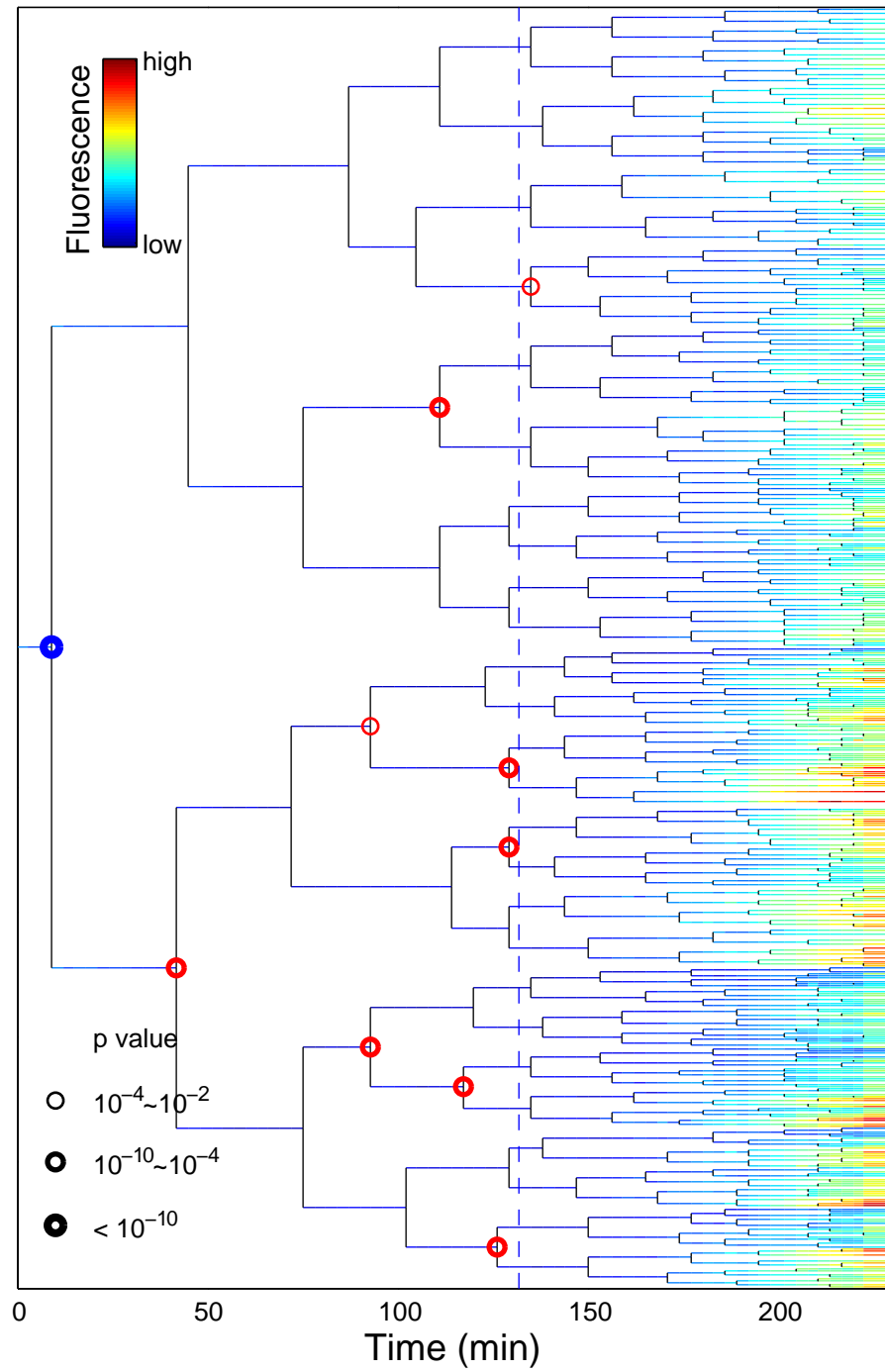

Supplement: Figure S12 — Correlation between progenitor sister cells fluorescence and progenies fluorescence intensity. The lineage tree corresponds to Video S3. Experimental condition is the same as Figure S11. (PDF) [file pgen.1003148.s012.pdf]

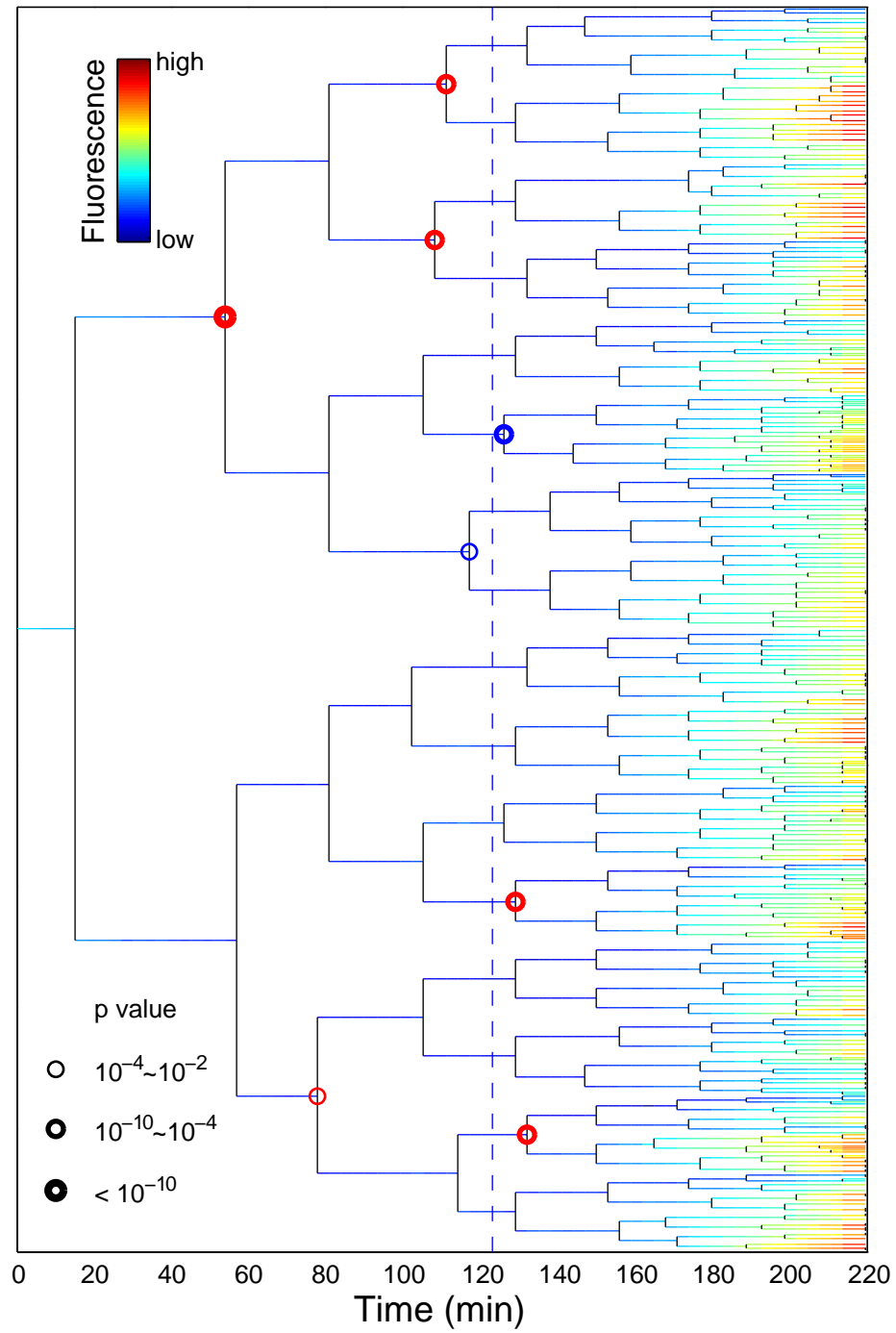

Supplement: Figure S13 — Correlation between progenitor sister cells fluorescence and progenies fluorescence intensity. Experimental condition is the same as Figure S11. (PDF) [file pgen.1003148.s013.pdf]

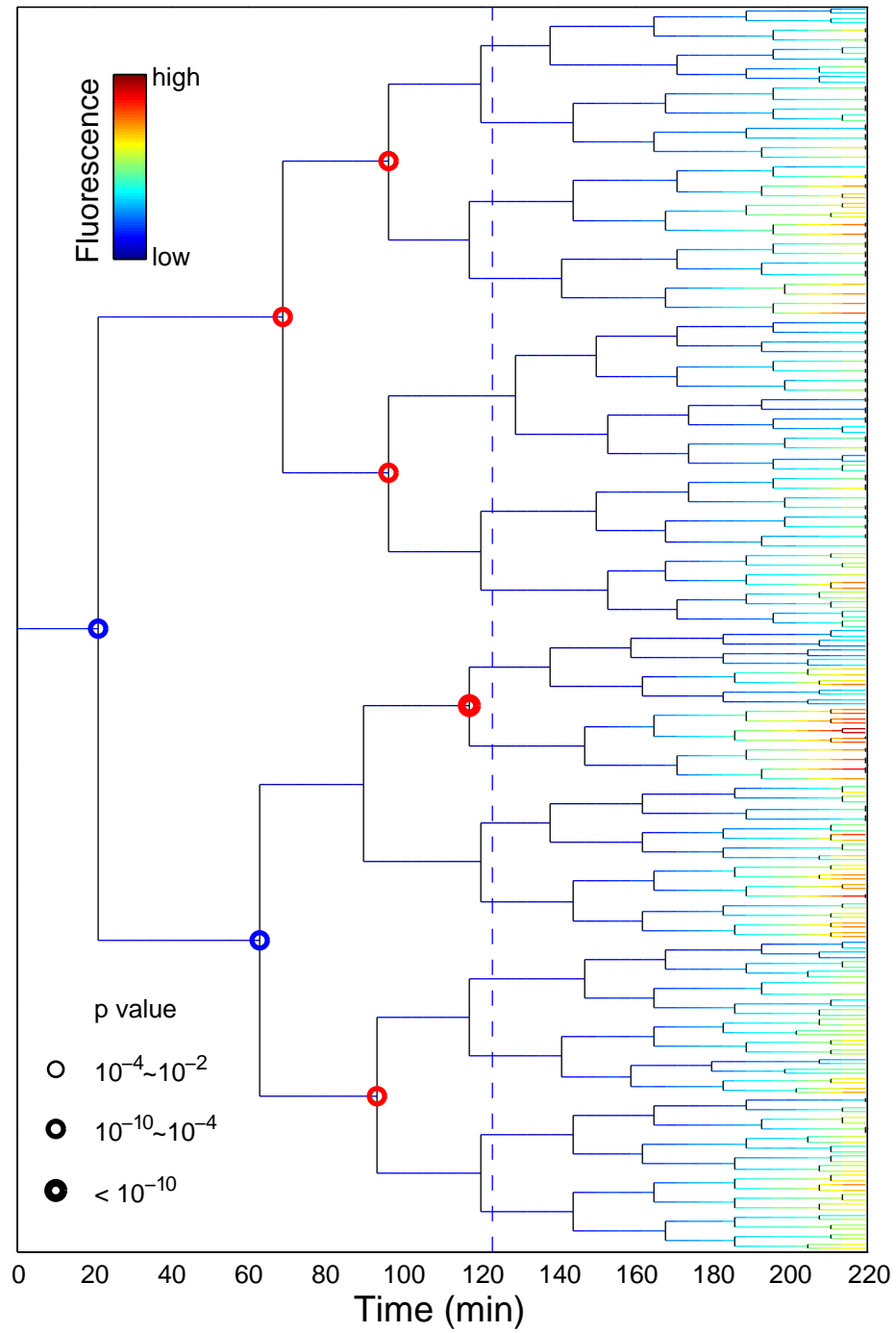

Supplement: Figure S14 — Correlation between progenitor sister cells fluorescence and progenies fluorescence intensity. Experimental condition is the same as Figure S11. (PDF) [file pgen.1003148.s014.pdf]

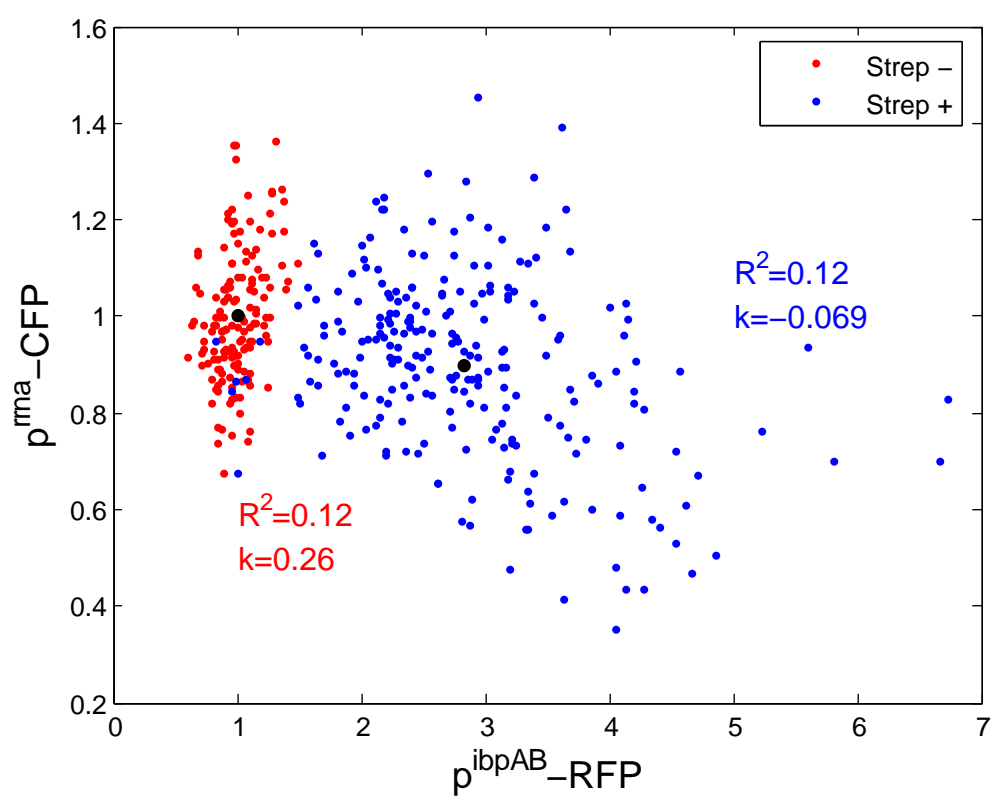

Supplement: Figure S15 — Correlation between pipbAB promoter and prrna promoter activity. Overnight culture is diluted 200 fold in LB in 37°C for 2 hours. The exponential phase cell culture is then diluted into LB medium with or without streptomycin for another 2 hour. Cells are then plated on agar pad and quantified under fluorescence microscope. Red and blue dots indicate non-induced and induced cells respectively. Fluorescence levels are normalized by the mean value of non-induced cells. The black dots indicate the mean value. (PDF) [file pgen.1003148.s015.pdf]

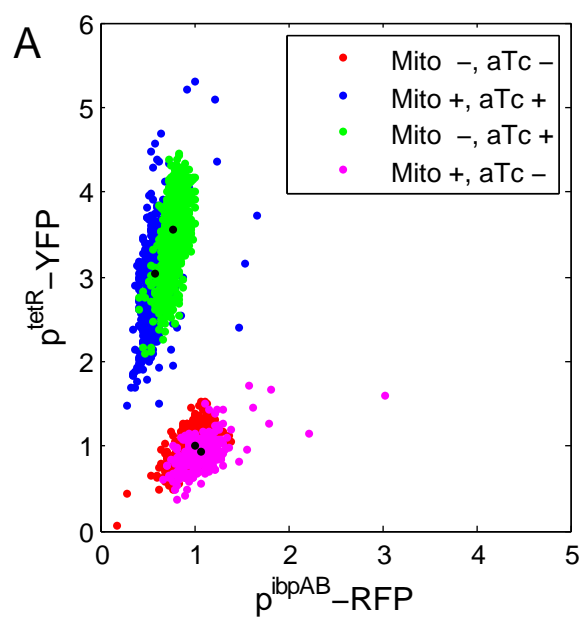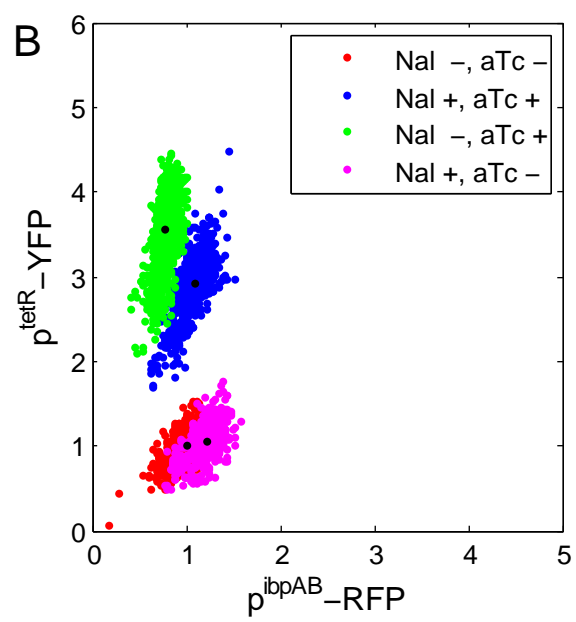

Supplement: Figure S16 — Co-induction of antibiotics and aTc. Exponential phase cells of strain harbouring both pterR and pibpAB driven fluorescence reporters are plated onto LB-agar pads containing ATC (25 ng/ml) with or without (A) Mitomycin (0.3 µg/ml) and (B) Nalidixic Acid (0.6 µg/ml). After 2–3 hours of colony growth, the fluorescence intensity is quantified under fluorescence microscope. The fluorescence intensity is normalized by the non-induced state ([mitoycin] = [nalidixic acid] = [ATC] = 0). The black dot in the middle of each data cloud shows the mean value of both fluorescence channels. For each condition, at least 5 micro-colonies are quantified. (PDF) [file pgen.1003148.s016.pdf]

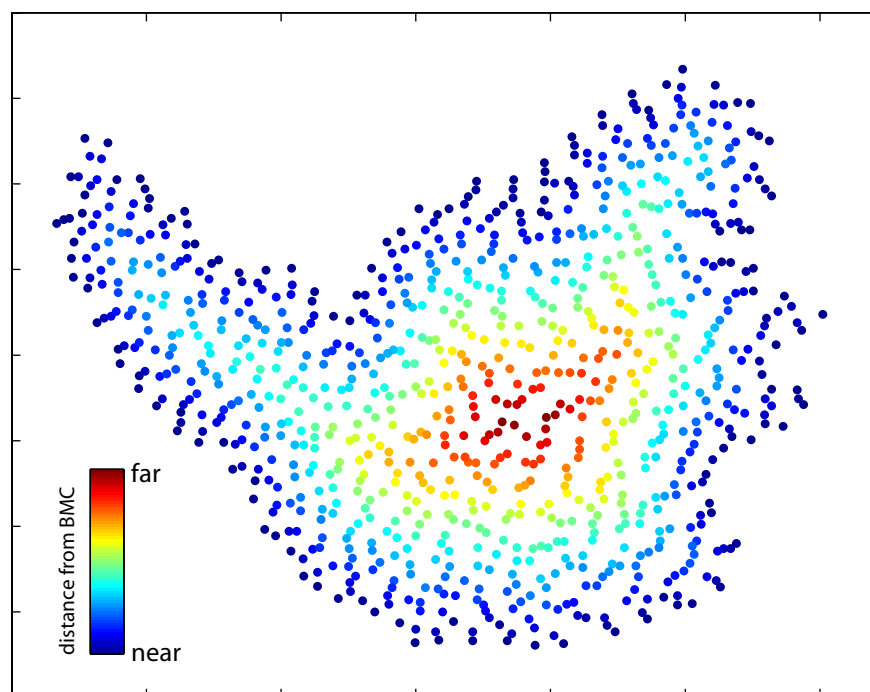

Supplement: Figure S17 — Cellular distance from micro-colony border. The distance between a single cell and the boarder of the micro-colony (BMC) is calculated as follows. Cells that are at the BMC are identified as boarder cells. The distance between boarder cells to BMC is zero. For the non-boarder cells, the distance of the cell to BMC is defined as the minimum distance between this cell to every boarder cells (pixel distance between cell mass centre). In this way, as long as the geometry of the micro-colony is not too far from convex polygon, which is mostly the case in our experiments, such definition is a good approximate of the geographical state of cells. In this figure, the dots represent mass centre of each cell. The colour denotes the BMC value. This is extracted from the last image of Video S2. (PDF) [file pgen.1003148.s017.pdf]

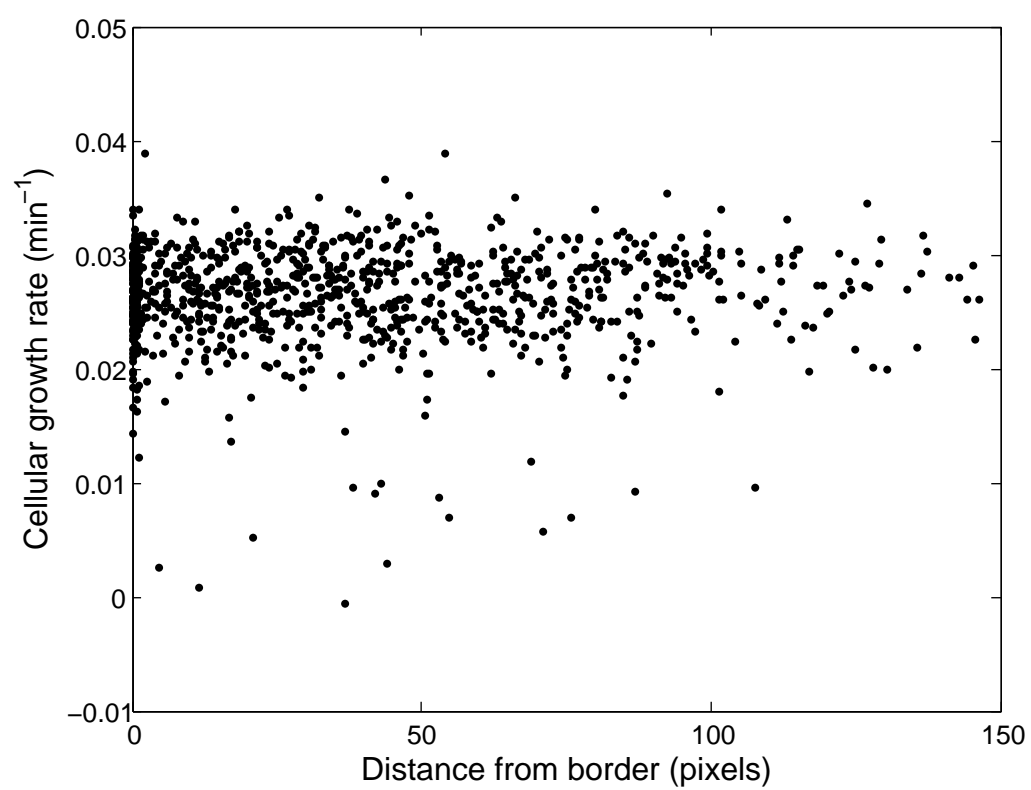

Supplement: Figure S18 — Correlation between cellular growth rate and their geographic location. The cellular growth rate is found to be independent of the distance of the cell to the boarder of micro-colony. Four micro-colonies (Figures S11, S12, S13, S14) are quantified at the last images of the time-lapsed movies. (PDF) [file pgen.1003148.s018.pdf]

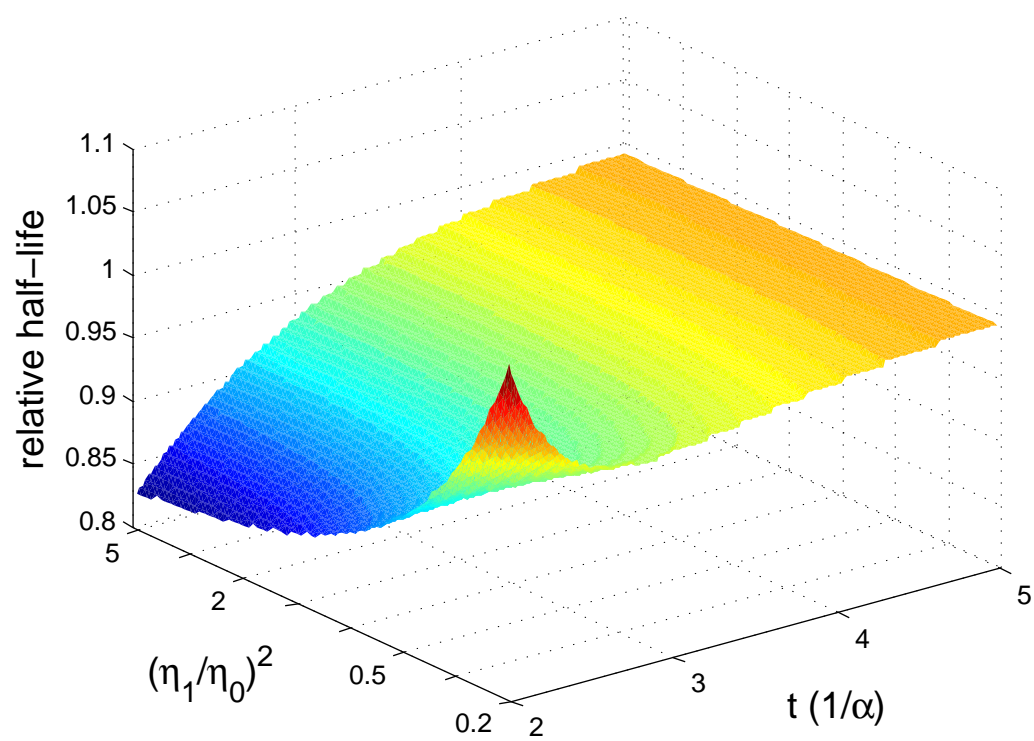

Supplement: Figure S19 — Autocorrelation half-life determined by the linear model. The autocorrelation half-life (relative to the cellular doubling time) is calculated according to equation (4) for different values of and t. (PDF) [file pgen.1003148.s019.pdf]
