# Supplementary material for: Pre-Disposition and Epigenetics Govern Variation in Bacterial Survival upon Stress
Source: PLoS Genet. 2012 Dec 20;8(12):e1003148. doi: 10.1371/journal.pgen.1003148 (PMC3527273; doi:10.1371/journal.pgen.1003148)
Supplement: Text S1 — Supplementary Methods. (PDF) [file pgen.1003148.s020.pdf]

Text S1

Supplementary Methods

- Sub-lineages coefficient of variation (SLCV) and individual cells coefficient of variation (IDCV)
- Autocorrelation function for cell lineage (AFCEL)
- Autocorrelation function at non-steady state
- Stochastic Differential Equations Model
- References

Sub-lineages coefficient of variation (SLCV) and individual cells coefficient of variation (IDCV)

Consider micro-colony originated from one single cell. At time s, the micro-colony reaches N_s cells. At later time point $t>s$, each cell in time s had produced n_i progenies respectively, whose fluorescence intensity or growth rate are denoted as x_i^k (i=1~Ns; k=1~n_i). Therefore, the total number of cells at time t is

$$N_t = \sum_{i=1}^{Ns} n_i$$

The averaged fluorescence intensity among cells within the same sub-lineage is

$$\overline{x_i} = \frac{1}{n_i}\sum_{k=1}^{n_i} x_i^k$$

We define the average of $\overline{x_i}$ as μ_s

$$\mu_s = \frac{1}{N_s}\sum_{i=1}^{N_s}\overline{x_i}$$

The SLCV for starting point s and end point t is then calculated as

$$SLCV(s,t) = \frac{\sqrt{\frac{N_t}{N_s}}\sqrt{\frac{1}{N_s}\sum_{i=1}^{N_s}(\overline{x_i}-\mu_s)^2}}{\mu_s}$$

Let IDCV be the coefficient of variation among all individual cells in the micro-colony.

$$IDCV(t) = \frac{\sqrt{\frac{1}{N_t}\sum_{i=1}^{N_s}\sum_{k=1}^{n_i}(x_i^k - \mu_I)^2}}{\mu_I}$$

Where

$$\mu_I = \frac{1}{N_t}\sum_{i=1}^{N_s}\sum_{k=1}^{n_i} x_i^k$$

To derive the relation between SLCV and IDCV, we consider a simplified condition when $n_i=n$ is a constant. (In case n_i is not a constant but with small variation, the same principle still holds.) Therefore

$$\mu_I = \mu_s = \mu$$

$$SLCV(s,t)$$

$$= \frac{\sqrt{\frac{n}{N_s}\sum_{i=1}^{N_s}(\overline{x_i} - \mu)^2}}{\mu}$$

$$= \frac{\sqrt{\frac{n}{N_s}\sum_{i=1}^{N_s}(\frac{1}{n}\sum_{k=1}^{n} x_i^k - \mu)^2}}{\mu}$$

$$= \frac{\sqrt{\frac{1}{nN_s}\sum_{i=1}^{N_s}(\sum_{k=1}^{n} x_i^k - n\mu)^2}}{\mu}$$

$$= \frac{\sqrt{\frac{1}{nN_s}\sum_{i=1}^{N_s}\left[\sum_{k=1}^{n}(x_i^k - \mu)\right]^2}}{\mu}$$

$$= \frac{\sqrt{\frac{1}{nN_s}\sum_{i=1}^{N_s}\left[\sum_{k=1}^{n}(x_i^k - \mu)^2 + \sum_{k=1}^{n}\sum_{l=1, l\neq k}^{n}(x_i^k - \mu)(x_i^l - \mu)\right]}}{\mu}$$

On the other hand,

$$IDCV(t) = \frac{\sqrt{\frac{1}{nN_s}\sum_{i=1}^{N_s}\sum_{k=1}^{n}(x_i^k - \mu)^2}}{\mu}$$

Compare the expression of SLCV and IDCV, the difference is in the term

$$\sum_{k=1}^{n}\sum_{l=1,l\neq k}^{n}(x_i^k - \mu)(x_i^l - \mu) \quad (*)$$

If there is no differenciation occurs among the sub-lineages, for each sub-lineage, we have

$$\overline{x_i} \approx \mu$$

In such case, the term (*) will be close to zero. Therefore

$$SLCV(s,t) = IDCV(t)$$

Otherwise, if differenciation occurs so that $\overline{x_i}$ deviate from μ, (*) will be larger than zero. Then

$$SLCV(s,t) > IDCV(t)$$

Autocorrelation function for cell lineage (AFCEL)

Consider micro-colony originated from one single cell. At time s, the micro-colony reaches N_s cells whose fluorescence intensity are denoted as x_i (i=1~Ns). At later time point t>s, each cell in time s had produced n_i progenies respectively, whose fluorescence intensity is denoted as x_i^k (i=1~Ns; k=1~n_i).

AFCEL is defined as

$$\mathrm{AFCEL}(t,s) = \frac{\frac{1}{N_t}\sum_{i=1}^{N_s}\sum_{k=1}^{n_i}(x_i - \overline{x_s})(x_i^k - \overline{x_t})}{\frac{1}{N_s}\sum_{i=1}^{N_s}\left(x_i - \overline{x_s}\right)^2}$$

Where

$$\overline{x_s} = \frac{1}{N_s}\sum_{i=1}^{N_s} x_i$$

$$\overline{x_t} = \frac{1}{N_t}\sum_{i=1}^{N_s}\sum_{k=1}^{n_i} x_i^k$$

Autocorrelation function at non-steady state

We examine how the autocorrelation function will be in the simplest linear model, without feedback controls. Let x be the concentration of protein expression level in the cell. The normalized autocorrelation function between time t and $t-\tau$ ($t>\tau$) is defined as

$$R(t,\tau)=\frac{<\tilde{x}(t)\tilde{x}(t-\tau)>}{\sqrt{<\tilde{x}^2(t)><\tilde{x}^2(t-\tau)>}}\qquad \tilde{x}(t)=x(t)-<x(t)>$$

Consider x is controlled by a constant production rate β and constant dilution rate α due to cellular growth. The dynamics of x can be described as

$$\frac{dx}{dt}=\beta-\alpha x+\eta(t)\qquad (1)$$

Where $\eta(t)$ is the intrinsic noise satisfying

$$<\eta(t)>=0;\quad <\eta(t)\eta(t')>=\eta^2\delta(t,t')$$

When the system reaches equilibrium, for any τ and t, there will be

$$<x(t)>=<x(t-\tau)>;\quad <x^2(t)>=<x^2(t-\tau)>$$

In this case the autocorrelation function does not depend on t and decades exponentially with τ. The half life of decay is $\ln 2/\alpha$, which equals to the cellular doubling time.

If the system is not in equilibrium state, for example at the beginning of stress induction, applying the similar deduction procedure as in (1), $R(t,\tau)$ is calculated as follows.

Consider the system had reached equilibrium before time t=0. At time =0, the production rate increased from β to β_1 ($\beta<\beta_1$). The intrinsic noise η also change to η_1. Therefore, if there is not feedback mechanism, the induction process (t>0) is described as

$$\frac{dx}{dt}=\beta_1-\alpha x+\eta_1(t)\quad (t>0)\qquad (2)$$

Let the equilibrium state of equation (1) be the initial condition of equation (2)

$$x(0)=\frac{1}{\alpha}(\beta+\eta_0)$$

Where η_0 is random noise.

$$<\eta_0>=0;\quad <\eta_0^2>=\eta_0^2$$

Solve equation (2)

$$x(t)=\left\{\int_0^t[\beta_1+\eta_1(t')]e^{\alpha t'}dt'\right\}e^{-\alpha t}+\left(\frac{\beta}{\alpha}+\frac{\eta_0}{\alpha}\right)e^{-\alpha t}\qquad (3)$$

According to (3),

$$< x(t) >=< \left\{ \int_0^t \beta_1 e^{\alpha t'} dt' \right\} e^{-\alpha t} + \left\{ \int_0^t \eta_1(t') e^{\alpha t'} dt' \right\} e^{-\alpha t} > + \frac{\beta}{\alpha} e^{-\alpha t}$$

$$= \left\{ \int_0^t \beta_1 e^{\alpha t'} dt' \right\} e^{-\alpha t} + \frac{\beta}{\alpha} e^{-\alpha t}$$

$$= \frac{\beta_1}{\alpha}(1 - e^{-\alpha t}) + \frac{\beta}{\alpha} e^{-\alpha t}$$

$$< x^2(t) >= \frac{e^{-2\alpha t}}{\alpha^2} \left[(\beta_1{}^2 + \eta_1{}^2)(e^{\alpha t} - 1)^2 + 2\beta\beta_1(e^{\alpha t} - 1) + \beta^2 + \eta_0{}^2 \right]$$

$$< x(t)x(t-\tau) >= \frac{e^{-2\alpha t + \alpha\tau}}{\alpha^2} [\beta_1{}^2 (e^{\alpha t} - 1)(e^{\alpha t - \alpha\tau} - 1) + \eta_1{}^2 (e^{\alpha t - \alpha\tau} - 1)^2$$
$$+ \beta\beta_1(e^{\alpha t} - 1) + \beta\beta_1(e^{\alpha t - \alpha\tau} - 1) + \beta^2 + \eta_0{}^2]$$

Because

$$< \tilde{x}^2(t) >=< (x(t) - < x(t) >)^2 >=< x^2(t) > - < x(t) >^2$$
$$< \tilde{x}(t)\tilde{x}(t-\tau) >=< x(t)x(t-\tau) > - < x(t) >< x(t-\tau) >$$

Therefore,

$$R(t,\tau) = \sqrt{\frac{\eta_1{}^2 (e^{\alpha t - \alpha\tau} - 1)^2 + \eta_0{}^2}{\eta_1{}^2 (e^{\alpha t} - 1)^2 + \eta_0{}^2}} = \sqrt{\frac{\sigma(e^{\alpha t - \alpha\tau} - 1)^2 + 1}{\sigma(e^{\alpha t} - 1)^2 + 1}} \qquad (4)$$

Where

$$\sigma = \frac{\eta_1{}^2}{\eta_0{}^2}$$

As shown in Figure S19, only when $\eta_0 >> \eta_1$ the autocorrelation half-life is longer than the doubling time $\ln(2)/\alpha$. This condition is in contrast to the increased variation after induction we observed (Figure 2). Furthermore, in Figure S19, as *t* increases, the autocorrelation half-life converges to the cellular doubling time, while in reality the autocorrelation half-life can be much longer than doubling time after induction (Figure 4). Therefore, we concluded that the simple linear model described by equations (1) and (2) is not able to explain the prolonged autocorrelation half-life. There should be nonlinear mechanisms like positive feedback that govern the induction process.

Stochastic Differential Equations Model

A stochastic differential equation model was built to describe stress-induced reporter gene expression and cell division. We take into account the positive feedback between stress level and reporter gene expression level. The reporter gene

expression is also inversely correlated with the cellular growth rate. The model is based on equation (1) while the feedback mechanisms are added on to β and α

$$x(i) = x(i-1) + (\beta - \alpha \cdot x(i-1) + \eta) \cdot \Delta t$$

$$l(i) = l(i-1)(\alpha + \sigma) \cdot \Delta t + l(i-1)$$

Where $x(i)$ is the reporter gene expression at time i and $l(i)$ is the cell length at time i. η and σ are Gaussian noise. In non-stressed condition, β and α are constants. In stressed condition, the promoter activity β positively correlate with expression level $x(i)$ while the cellular growth rate α negatively correlate with it.

$$\beta(i) = 1 + \frac{a}{1 + \left(\frac{b}{x(i)}\right)^n}$$

$$\alpha(i) = \frac{1}{1 + \frac{x(i)}{c}}$$

The initial cell length l is normalized as 1. When the cell length increases to 2, one cell divides into two. The two daughter cells' initial cell lengths are set to be 1 again and they inherit the mother's β and α value. Model parameters were set to fit the mean and variance of single cell phenotypes measured from the experimental data.

As shown in Figure S7, in agreement with our experimental observations, the simulation results demonstrate that IDCV and SLCV are at the same level in normal, non-stressed condition, while the extended cell memory effect has led to significantly increased SLCV in the stress response regime.

Reference

1. Sigal A, *et al.* (2006) Variability and memory of protein levels in human cells. *Nature* 444(7119):643-646.
